# Trends estimation of obesity prevalence among South Asian young population: a systematic review and meta-analysis

**DOI:** 10.1038/s41598-023-50973-w

**Published:** 2024-01-05

**Authors:** Dipika Bansal, Mohammed Safeer V. S., Nagita Devi, Chandrasekhar Boya, Karamsetty Dhora Babu, Pinaki Dutta

**Affiliations:** 1 Department of Pharmacy Practice, National Institute of Pharmaceutical Education and Research (NIPER), S.A.S. Nagar, Punjab 160062 India; 2 Department of Endocrinology, Postgraduate Institute of Medical Education and Research (PGIMER), Chandigarh, India

**Keywords:** Endocrine system and metabolic diseases, Epidemiology

## Abstract

The premise for effective prevention and treatment of obesity is the availability of accurate prevalence figures. However, the prevalence of pediatric obesity and overweight in South Asian countries has seldom been analyzed. This article provides a comprehensive review and meta-analysis of studies on overweight and obesity to provide a more precise prevalence estimate. The study protocol was registered on PROSPERO (CRD42022320625). PubMed and Embase databases were comprehensively searched from inception till September 2023. The random-effects model was utilized to derive the pooled prevalence of obesity and overweight. Subgroup meta-analysis was used to assess variations in prevalence estimates across subgroups. A meta-regression analysis was also performed to assess the trend of overweight and obesity over the years. 152 studies were included with 489,525 participants. The pooled prevalence was 12.4 (95% CI 11.1–13.6) for overweight, 6.6% (95% CI 5.6–7.8) for obesity, and 19.3% (95% CI 17.1–21.7) for obesity and overweight. In subgroup analysis, Bangladesh reported a higher prevalence for both obesity (8.9%; 95% CI 4.9–13.9) and overweight (13.6%; 95% CI 9.2–18.8). Meta-regression analysis found a significant association between obesity prevalence and the publication year (β = 0.004; p = 0.03; R^2^ = 2.74%). The results of this study indicate a relatively higher prevalence of childhood obesity in South Asia, emphasizing the necessity for large-scale awareness efforts and context-specific preventative methods.

## Introduction

Globally, a huge proportion of the population is affected by obesity (OB)/overweight (OW) which contributes to the development of non-communicable diseases (NCDs), regardless of age, gender, race/ethnicity^1,2^. In recent years, the prevalence of OB/OW in children and adolescents has gained considerable attention. This is due to the fact that childhood and adolescence are the formative years during which individuals establish the foundation for their future health. In addition, being obese throughout this period of life increases the likelihood of continuing it throughout their lifetime^3^.

It is believed that more than 90% of cases are caused by modifiable variables such as unhealthy eating habits, disrupted sleep patterns, and inadequate physical activity. Conversely, a mere 10% of cases are thought to arise from hormonal or genetic alterations^4^. OB is also strongly associated with growing medical expenses and as per the estimates, the economic cost of OB worldwide in 2014 was $2.0 trillion, or 2.8% of the global gross domestic product (GDP)^5^. In addition to the healthcare expenses, OB levies cost in the form of stalled economic growth due to missed days at work, decreased productivity, death, and permanent disability^6^. According to the World Health Organization (WHO), the prevalence of OB and OW among adolescents and young adults has increased dramatically from 4% in 1976 to over 18% in 2016. This trend was observed in both boys and girls, with 19% of boys and 18% of girls being OW^7^. In a global systematic analysis (1980–2013), South Asian countries such as India, Nepal, Bangladesh, Bhutan, and Pakistan reported OB prevalence rates of 2.4%, 1.9%, 1.5%, 5.8%, and 3.9%, respectively^8^. A systematic review also reported that the prevalence of OB and OW in Indian children between 2010 and 2013 was 19.3%, a considerable rise from the previous prevalence of 16.6% recorded between 2001 and 2005^9^.

However, contemporary data on the prevalence of OB and OW across South Asian countries are relatively sparse. A comprehensive systematic review of OW and OB prevalence particularly in South Asian countries^10^ is required to determine the burden of OW/OB and to develop region-specific prevention strategies aimed at the management and prevention of OB in this region. Hence, our systematic review and meta-analysis aimed to estimate the prevalence of OW and OB in the younger population of South Asian countries.

## Materials and methods

This study was registered in PROSPERO (CRD42022320625) following the Preferred Reporting Items for Systematic Reviews and Meta-Analyses (PRISMA) guidelines.

### Study eligibility

Cross-sectional studies from South Asian nations that had reported the prevalence of OB and OW (as per standard international or national criteria like WHO^11,12^, International Obesity Task Force (IOTF)^13^, Indian Academy of Paediatrics (IAP)^14^, and Centre for Disease Control (CDC)^15^ classification systems) in children aged ≤ 19 years were included. Reviews, randomized controlled trials, case–control studies, case reports, comments, letters, pilot studies, conference abstracts or posters, and other research lacking original data were excluded. Studies that measured OB and OW as per mid-upper arm circumference and waist-to-hip ratio were also not eligible for this study.

### Searches

PubMed and Embase databases were systematically searched utilizing the keywords like “adolescents”, “obesity”, “overweight”, “children”, “body mass index”, and “South Asia” from inception till September 2023. The search strategy followed for the database search is detailed in Supp. Table 1. To prevent excluding any possibly pertinent research, Google Scholar and references of the publications were also thoroughly searched. No restriction was imposed on language or publication date.

### Study selection and data extraction

After the elimination of duplicates, each retrieved citation was screened based on the title and abstracts for study eligibility by two independent review authors (MS&ND). The complete text of the relevant citations was assessed critically, as per pre-defined eligibility criteria before proceeding with the data extraction of all relevant information such as author, publication year, country, timeframe, sample size, sampling technique, sample population, study design, diagnostic criteria, mean age, gender, and prevalence data for OB/OW. Any inconsistencies or disputes that arose during study selection and the extraction process were resolved by discussion between two review authors (MS&ND) and, if necessary, a third review author (DB).

### Study quality assessment

The critical quality appraisal of the individual study was performed by two independent review authors (MS&ND) with the Joanna Briggs Institute (JBI) checklist for prevalence studies^16^. The JBI appraisal checklist consists of nine elements, scored from zero to nine. Each study’s total score was classified into three levels (high risk if 0–50% of items were answered yes, moderate risk if 50–80% of items were answered yes, and low risk if 80–100% of items were answered yes)^17^.

### Overweight and obesity diagnosis in the included studies

The included studies assessed the children/adolescents for OW and OB using the following international and national classifications: IOTF^13^, WHO^11,12^, IAP^14^, CDC^15^, National Centre for Health Statistics (NCHS)^18^, Eliz Health Path for Adolescents and Adults (EHPA)^19^, Agarwal et al.^20^ and Rosner et al.^21^ criteria.

### Statistical analysis

A random-effect meta-analysis (DerSimonian and Laird) was performed using Freeman–Tukey double arcsine transformed proportion^22^. The random effect model was applied throughout the study due to the expected heterogeneity between the studies. The extent of heterogeneity was evaluated by using the I^2^ statistic as no heterogeneity (0%), low (≤ 25%), moderate (25–50%), substantial (50–75%), and high (≥ 75%)^23,24^. Subgroup meta-analysis was used to assess variations in prevalence estimates across various study characteristics^25^. Qualitative and quantitative identification of publication bias was conducted using the visual examination of funnel plots and the Eggers test, respectively^26,27^. The trim-and-fill method of Duval and Tweedie was used to adjust the publication bias for the pooled estimates^28^. Leave-1-out sensitivity analysis was conducted to evaluate the impact of the individual study on the pooled estimates^29^. A meta-regression analysis was performed to explore the trend of the prevalence of OW and OB over time. All statistical analyses were performed using the meta package in R (version 4.2.3).

## Results

### Search results

**Figure 1:**
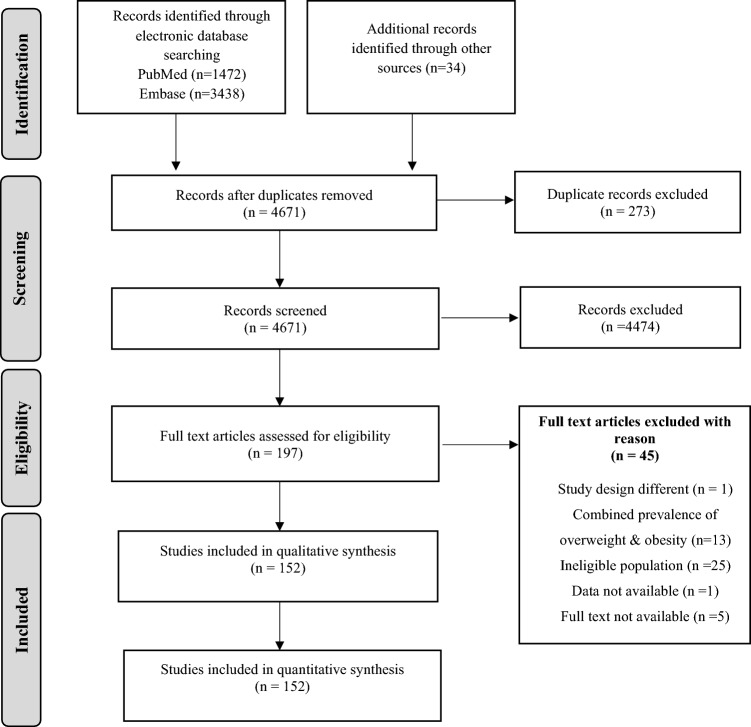
PRISMA flowchart.

After the removal of 273 duplicates, 4671 citations were screened for the title and abstracts as per pre-defined eligibility criteria. Of them, 197 potentially relevant citations were screened for full text. Finally, 152 studies (Supp. Information for reference list) were included in this systematic review and meta-analysis (Fig. 1).

### Study characteristics

The baseline study characteristics are detailed in Supp. Table 2. A total of 152 studies involving 489,525 children/adolescents had reported the prevalence of OW/OB/OW + OB from the South Asian regions and were published between 1994 and 2023. Among them, 112 (74%) studies were performed in India, 17 (11.2%) in Pakistan, 10 (7%) in Bangladesh, 9 (6%) in Nepal, 2 (1.3%) in Sri Lanka, and one (0.6%) in Bhutan and Maldives. 130 studies reported the prevalence of both OB and OW while 18 studies reported the OW prevalence and only 4 studies reported the prevalence of OB. Of the included studies 99 (65.1%) studies were performed in an urban setting, 9 (6%) studies were performed in a rural setting and 44 (29%) studies were performed in a mixed setting. Among the included studies, 132 (87%) were school-based, 9 (6%) were community-based, and 11 (7.2%) were hospital-based studies. In 41 (27%) studies, WHO (2007) criteria was used to diagnose OW and OB, whereas CDC criteria was used in 29 (19.1%) studies, IOTF criteria in 17 (11.1%), IAP criteria in 16 (10.5%), WHO (2006) criteria in 5 (3.2%), NCHS criteria in 3 (2%) studies, WHO (1995) criteria in 2 (1.3%) and EHPA, Rosner et al. and Agarwal et al. criteria were used in one study. For diagnosis, 19 (12.5%) studies used multiple criteria while 17 studies (11.18%) did not mention the diagnostic criteria used. The participants included in the study were between 0 to 19 years old.

### Risk of bias

Using JBI’s critical appraisal tool for prevalence studies, 86 studies were found to have a low risk, whereas 66 studies had a moderate risk. Among the included studies, the sampling procedure was not specified in 38 studies, 37 studies did not have an acceptable sample size and 13 studies did not use a standard method for the identification of the condition. The mean quality (SD) score was found to be 86.04% (11.85) (Supp. Table 3).

### Prevalence of OW/OB

The pooled prevalence of OW was 12.4% (95% CI 11.1–13.6; number of studies (N): 148; I^2^: 99%), while the prevalence of OB was 6.6% (95% CI 5.6–7.8; N: 135; I^2^: 99%) in children at the South Asian region. Moreover, the prevalence of OB + OW was found to be 19.3% (95% CI 17.1–21.7; N: 131; I^2^: 100%). Table 1 summarizes the subgroup analysis of both OW and OB.

**Table 1:** Prevalence of OB/OW in different subgroups.

Study characteristics	Obesity			Overweight			Overweight + obesity		
	No of studies included	No of cases/total participants	Prevalence (95% CI)	No of studies included	No of cases/total participants	Prevalence (95% CI)	No of studies included	No of cases/total participants	Prevalence (95% CI)
All studies	135	25,947/487,609	6.6 (5.6–7.8)	148	52,302/415,824	12.4 (11.1–13.6)	131	74,759/413,908	19.3 (17.1–21.7)
Country									
India	102	23,497/438,349	6.5 (5.3–7.9)	109	47,635/368,015	12.7 (11.2–14.2)	99	67,972/367,848	19.3 (16.6–22.2)
Pakistan	17	1221/27,659	7.1 (4–11)	16	2071/24,459	11 (7.8–14.7)	16	3139/24,459	19 (12.7–26.2)
Bangladesh	8	901/16,029	8.9 (4.9–13.9)	10	1812/16,487	13.6 (9.2–18.8)	8	2642/16,029	23.4 (14.6–33.5)
Nepal	5	226/3248	5.9 (2.3–11.1)	9	534/4394	10.1 (5–16.9)	5	674/3248	19.4 (11.6–28.6)
Sri Lanka	2	96/1932	4.8 (0.0–35.8)	2	202/1932	10.4 (3.8–19.9)	2	298/1932	15.3 (0.1–48.1)
Bhutan	1	6/392	1.5 (0.5–3)	1	28/392	7.1 (4.8–9.9)	1	34/392	8.7 (6.1–11.7)
Maldives	NR	NR	NR	1	20/145	13.8 (8.6–19.9)	NR	NR	NR
Diagnostic criteria									
WHO 2007	46	9578/196,187	5.9 (4.5–7.4)	55	17,830/137,571	12.6 (10.3–15)	45	25,856/128,446	18.6 (15.7–21.6)
CDC	33	4284/86,414	6.1 (3.5–9.4)	35	6835/87,586	9.6 (7.9–11.5)	33	10,991/86,414	17.1 (11.3–23.8)
IOTF	32	7319/196,057	4.0 (3.2–4.9)	36	25,356/202,850	11.9 (10.1–13.9)	32	31,321/196,057	15.7 (13.1–18.4)
IAP	22	5773/59,353	9.2 (7–11.7)	23	10,641/57,501	15.2 (11.9–18.9)	22	16,759/59,353	25.2 (19.5–31.4)
Agarwal et al.	5	2245/63,711	6.5 (1.2–15.4)	6	6983/67,415	14.2 (6.3–24.5)	5	8009/63,711	18.2 (8.3–30.8)
NCHS	2	373/7402	5.0 (2.6–8.3)	1	37/202	18.3 (13.3–24)	1	380/4202	9.0 (8.2–9.9)
WHO 2006	2	30/597	6.1 (0–100)	5	92/1494	6.2 (0.1–19.7)	2	93/597	19.8 (0.0–100)
WHO 1995	1	27/3356	0.8 (0.5–1.1)	3	437/4961	12.5 (0.01–41.5)	1	168/3356	5.0 (4.3–5.8)
Rosner et al.	1	34/1000	3.4 (2.4–4.6)	1	127/1000	12.7 (10.7–14.8)	1	161/1000	16.1 (13.9–18.4)
EHPA	1	222/2570	8.6 (7.6–9.8)	1	499/2570	19.4 (18–21)	1	721/2570	28.1 (26.3–29.8)
Study setting									
Urban	92	20,993/323,152	7.3 (6–8.9)	98	43,291/327,395	13.3 (11.9–14.7)	91	61,691/320,592	21.6 (18.5–24.7)
Rural	6	110/5512	3.0 (0.9–6.3)	9	521/6677	12.8 (2.8–28.6)	6	325/5512	10.2 (4–18.7)
Gender									
Male	80	11,417/177,483	6.6 (5.5–7.8)	86	22,718/177,344	13.6 (10.8–13.9)	78	32,660/180,642	18.2 (15.8–20.8)
Female	83	8164/169,159	6.1 (4.8–7.5)	93	19,479/170,817	12.5 (11–13.9)	80	25,189/158,265	17.5 (15–20.1)
Publication year									
Less than 2010	22	3243/98,431	4.4 (3–6)	23	10,289/100,728	12.3 (9.2–15.8)	21	11,962/95,871	16.1 (12–20.6)
2010–2013	25	9037/136,595	5.6 (4–7.4)	26	18,776/134,220	12.1 (9.6–14.7)	24	26,807/133,395	18 (14.3–22.1)
2014–2018	33	7549/104,710	7.5 (5.8–9.6)	34	14,035/105,143	12.8 (10.7–15)	33	21,543/104,710	21.1 (17.3–25)
2019–2023	55	6118/147,873	7.6 (5.4–10.4)	65	9202/7533	12.3 (10.2–14.5)	53	14,447/79,932	20.2 (15.6–25.2)
Sample size									
≤ 1000	69	23,121/454,498	7.6 (5.7–9.9)	81	5189/38,387	12.6 (10.8–14.5)	65	7154/32,911	21 (17–25.1)
> 1000	66	2826/33,111	5.8 (4.7–6.8)	67	47,113/377,437	12.1 (10.5–13.7)	66	67,605/380,997	17.8 (15.5–20.3)
Age group									
0–9	4	100/1613	6.3 (0.5–17.2)	9	278/3169	8.6 (3.2–16.1)	5	299/2046	15.8 (3.2–35.3)
10–19	61	6592/101,379	6.2 (4.5–8.1)	67	14,301/98,884	12.2 (10.6–14.0)	63	20,455/97,826	19.0 (15.2–23.1)

*WHO* World Health Organisation, *IOTF* International Obesity Task Force, *IAP* Indian Academy of Paediatrics, *CDC* Centre for Disease Control and Prevention, *NCHS* National Centre for Health Statistics, *EHPA* Eliz health path for adolescents and adults, *NA* not reported.

### Country-wise distribution of OW/OB

The country-wise distribution of OW was observed higher in children and adolescents who belonged to Bangladesh (13.6%; 95% CI 9.2–18.8; N: 10; I^2^: 95%) followed by India (12.7%; 95% CI 11.2–14.2; N: 109; I^2^: 99%), Pakistan (11%; 95% CI 7.8–14.7; N: 16; I^2^: 98%), Sri Lanka (10.4%; 95% CI 3.8–19.9; N: 2; I^2^: 0%) Nepal (10.1%; 95% CI 5–16.9; N: 9; I^2^: 96%), and Bhutan (7.1%; 95% CI 4.8–9.9; N: 1). Additionally, Maldives reported a higher OW prevalence of 13.8% (95% CI 8.6–19.9) with a single study.

Similarly, the prevalence of OB was higher in children and adolescents from Bangladesh (8.9%; 95% CI 4.9–13.9; N: 8; I^2^: 98%) followed by Pakistan (7.1%; 95% CI 4–11; N: 17; I^2^: 98%), India (6.5%; 95% CI 5.3–7.9; N: 102; I^2^: 99%), Nepal (5.9%; 95% CI 2.3–11.1; N: 5; I^2^: 93%), Sri Lanka (4.8%; 95% CI 0.0–35.8; N: 2; I^2^: 88%) and Bhutan (1.5%; 95% CI 0.5–3; N: 1).

Furthermore, the prevalence of OB + OW was also observed higher in children and adolescents from Bangladesh (23.4%; 95% CI 14.6–33.5; N: 8; I^2^: 99%) followed by Nepal (19.4%; 95% CI 11.6–28.6; N: 5; I^2^: 94%), India (19.3%; 95% CI 16.6–22.2; N: 99; I^2^: 100%), Pakistan (19%; 95% CI 12.7–26.2; N: 16; I^2^: 99%), Sri Lanka (15.3%; 95% CI 0.1–48.1; N: 2; I^2^: 84%) and Bhutan (8.7%; 95% CI 6.1–11.7; N: 1).

### Diagnostic criteria

The highest prevalence of OW in children and adolescents was observed using the EHPA (19.4%; 95% CI 18–21.0; N: 1) diagnostic criteria followed by NCHS (18.3%; 95% CI 13.3–24; N: 1), IAP (15.2%; 95% CI 11.9–18.9; N: 23; I^2^: 99%), Agarwal et al. (14.2%; 95% CI 6.3–24.5; N: 6; I^2^: 100%), Rosner et al. (12.7%; 95% CI 10.7–14.8; N: 1), WHO 2007 (12.6; 95% CI 10.3–15; N: 55; I^2^: 99%), WHO 1995 (12.5%; 95% CI 0.01–41.5; N: 3; I^2^: 99%), IOTF (11.9%; 95% CI 10.1–13.9; N: 35; I^2^: 99%), CDC (9.6%; 95% CI 7.9–11.5; N: 36; 98%) and WHO 2006 (6.2%; 95% CI 0.1–19.7; N: 5; I^2^: 96%).

However, the IAP (9.2%; 95% CI 7–11.7; N: 22; I^2^: 97%) diagnostic criteria have classified the highest proportion of children and adolescents with OB followed by EHPA (8.6%; 95% CI 7.6–9.8; N: 1), Agarwal et al. (6.5%; 95% CI 1.2–15.4; N: 5; I^2^: 99%), CDC (6.1%; 95% CI 3.5–9.4; N: 33; I^2^: 99%), WHO 2006 (6.1%; 95% CI 0–100; N: 2; I^2^: 97%), WHO 2007 (5.9%; 95% CI 4.5–7.4; N: 46; I^2^: 99%), NCHS (5.0%; 95% CI 2.6–8.3; N: 2; I^2^: 0%), IOTF (4%; 95% CI 3.2–4.9; N: 32; I^2^: 98%), Rosner et al. (3.4%; 95% CI 2.4–4.6; N: 1) and WHO 1995 (0.8%; 95% CI 0.5–1.1; N: 1).

Moreover, the EHPA (28.1%; 95% CI 26.3–29.8; N: 1) diagnostic criteria has classified the highest proportion of children and adolescents with OB + OW followed by IAP (25.2%; 95% CI 19.5–31.4; N: 22; I^2^: 99%), WHO 2006 (19.8%; 95% CI 0.0–100; N: 2; I^2^: 99%), WHO 2007 (18.6%; 95% CI 15.7–21.6; N: 45; I^2^: 99%), Agarwal et al. (18.2%; 95% CI 8.3–30.8; N: 5; I^2^: 99%), CDC(17.1%; 95%CI: 11.3–23.8; N: 33; I^2^: 100%), Rosner et al. (16.1%; 95% CI 13.9–18.4; N: 1), IOTF (15.7%; 95% CI 13.1–18.4; N: 30; I^2^: 99%), NCHS (9.0%; 95% CI 8.2–9.9; N: 1) and WHO 1995 (5.0%; 95% CI 4.3–5.8; N: 1).

### Study setting

An approximately similar prevalence of OW was observed across the urban (13.3%; 95% CI 11.9–14.7; N: 98; I^2^: 99%) and rural (12.8%; 95% CI 2.8–28.6; N: 9; I^2^: 99%) settings. While the prevalence of OB was observed higher in children and adolescents who belonged to urban (7.3%; 95% CI 6–8.9; N: 92; I^2^: 99%) settings as compared with rural (3.01%; 95% CI 0.9–6.3; N: 6; I^2^: 88%) settings. Likewise, the prevalence of OB + OW was observed higher in urban children and adolescents (21.6%; 95% CI 18.5–24.7; N: 91; I^2^: 100%) than in rural (10.2%; 95% CI 4–18.7; N: 6; 96%).

### Gender-wise distribution

The pooled prevalence of OW was observed higher in males (13.6%; 95% CI 12–15.4; N: 86; I^2^: 98%) than in females (12.5%; 95% CI 11.1–13.9; N: 93; I^2^: 98%). Moreover, the prevalence rate of OB and OW + OB were also observed to be higher in males (6.6%; 95% CI 5.5–7.8; N: 80; I^2^: 99% and 18.2%; 95% CI 15.8–20.8; N: 78; I^2^: 99% respectively) than in female children (6.1%; 95% CI 4.8–7.5; N: 83; I^2^: 98% and 17.5%; 95% CI 15–20.1; N: 80; I^2^: 99% respectively).

### Publication year

A total of 148 studies had reported the OW prevalence from inception to 2023. In subgroup analysis, the pooled OW prevalence was observed higher before 2010 (12.3%; 95% CI 9.2–15.8; N: 23; I^2^: 100%) followed by a decrease in 2010–2013 (12.1%; 95% CI 9.6–14.7; N: 26; I^2^: 99%) and increase in 2014–2018 (12.8%; 95% CI 10.7–15; N: 34; I^2^: 99%). From 2019 to 2023, there was a slight decrease in the prevalence of OW (12.3%; 95% CI 10.2–14.5; N: 65; I^2^: 99%).

In contrast, the prevalence of OB in children increased exponentially in recent years such as 4.4% (95% CI 3–6; N: 22; I^2^: 98%) before 2010, 5.6% (95% CI 4.0–7.4; N: 25; I^2^: 99%) between 2010 and 2013; 7.5% (95% CI 5.8–9.6; N: 33; I^2^: 99%) between 2014 and 2018, and 7.6% (95% CI 5.4–10.4; N: 55; I^2^: 99%) between 2019 and 2023.

The prevalence of OB + OW in children also increased exponentially with years like 16.1% (95% CI 12–20.6; N: 21; I^2^: 99%) before 2010, 18% (95% CI 14.3–22.1; N: 24; I^2^: 100%) between 2010 and 2013; 21.1% (95% CI 17.3–25; N: 33; I^2^: 99%) between 2014 and 2018. However, there was a slight decrease in prevalence between 2019 and 2023 (20.2%; 95% CI 15.6–25.2; N: 53; I^2^: 99%).

### Sample size

A total of 148 studies with sample sizes ranging from 100 to 43,152 reported the prevalence of OW in children. Of them, 81 studies had a sample size ≤ 1000 and reported an OW prevalence of 12.6% (95% CI 10.8–14.5; I^2^: 96%) while 67 studies reported a sample size > 1000.yielding an overall prevalence of 12.1% (95% CI 10.5–13.7; I^2^: 100%).

The prevalence of OB in children and adolescents was reported in 135 studies with sample sizes ≤ 1000 reported an overall prevalence of 7.6% (95% CI 5.7–9.9; N: 69; I^2^: 98%), while the sample size > 1000 reported an overall prevalence of 5.8% (95% CI 4.7–6.8; N: 69; I^2^: 100%).

The prevalence of OB + OW in children was reported in 131 studies with a sample size of ≤ 1000 reported a pooled prevalence of 21% (95% CI 17–25.1; N: 65; I^2^: 99%), while the studies with sample size > 1000 reported a prevalence of 17.8% (95% CI 15.5–20.3; N: 66; I^2^: 100%).

### Age group

The prevalence of OW and OB were separately pooled for children (0–9) and adolescents (10–19). A higher prevalence of OW was observed in adolescents (12.2%; 95% CI 10.6–14.0; N: 67; I^2^: 99%) than in children (8.6%; 95% CI 3.2–16.1; N: 9; I^2^: 96%). Similarly, a higher prevalence of OB + OW was observed in adolescents (19.0%; 95% CI 15.2–23.1; N: 63; I^2^: 99%) than in children (15.8%; 95% CI 3.2–35.3; N: 5; I^2^: 97%). However, the prevalence of OB was similar in both children (6.3%; 95% CI 0.5–17.2; N: 4; I^2^: 93%) and adolescents (6.2%; 95% CI 4.5–8.1; N: 4; I^2^: 99%).

### Publication bias and sensitivity analysis

**Figure 2:**
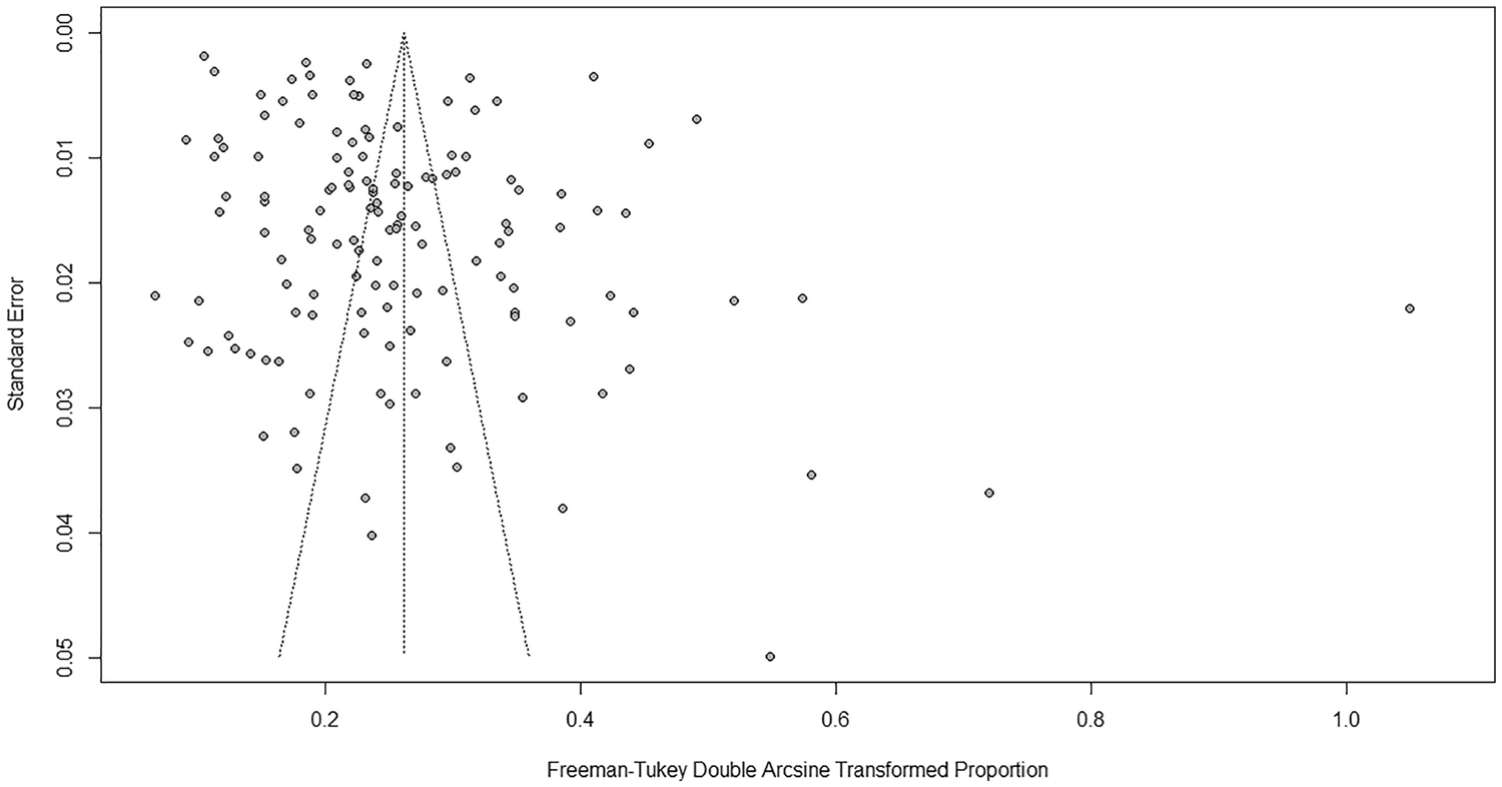
Funnel plot exhibiting publication bias for OB.

Publication bias was evident for the prevalence of OB (p-value = 0.0003 for Egger’s test; Fig. 2). The trim and fill method was used to adjust the publication bias and yielded a pooled OB prevalence of 3.2% (95% CI 2.3–4.2). The funnel plots and Eggers test demonstrated an absence of publication bias for both OW (p-value = 0.53; Supp. Fig. 1) and OW + OB (p-value = 0.13; Supp. Fig. 2) prevalence. Leave one out sensitivity revealed that no study had a significant impact on the pooled estimates of OB/OW. The pooled estimates ranged from 12.2 to 12.5% for OW, 6.5 to 6.7% for OB, and 18.3% to 19.5% for OW + OB.

### Meta-regression

**Figure 3:**
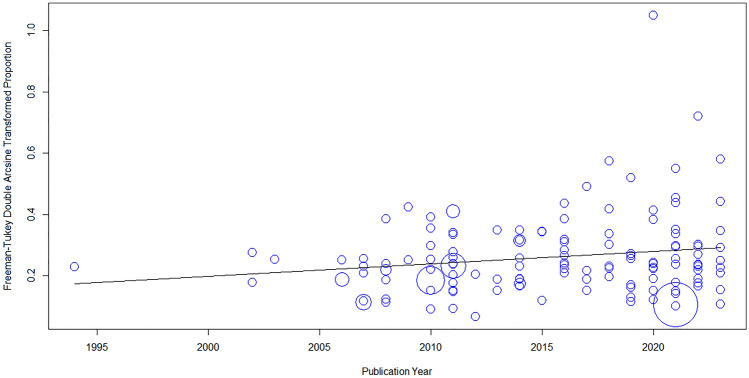
Bubble plot exhibiting association between publication year and prevalence of OB.

Meta-regression analysis showed that the prevalence of OB was significantly associated with the publication year of the included studies (β = 0.004; p-value = 0.03; R^2^ = 2.74%; Fig. 3). However, the prevalence of OW (β = 0.0002; p-value = 0.89; R^2^ = 0%; Supp. Fig. 3) and OB + OW (β = 0.003; p-value = 0.22; R^2^ = 0.4%; Supp. Fig. 4) were not significantly associated with the publication year.

## Discussion

To the best of our knowledge, this meta-analysis provides the most up-to-date and comprehensive evidence on the epidemiological burden of OB/OW in South Asian countries, summarizing estimates from 152 studies published from 1994 to 2023. The prevalence estimates for OB, OW, and OB + OW were found to be 6.6%, 12.4%, and 19.3% respectively. Children and adolescents in Bangladesh reported a higher prevalence of OW at 13.6%, whereas Bhutan reported a lower prevalence of 7.1%. Similarly, the prevalence estimates of OB also remained higher in children from Bangladesh. These results were consistent with the findings of a meta-analysis conducted by Biswas et al.^30^, which reported a pooled prevalence of 6% for OB^31^. In 2014, a national epidemiological survey among children aged 6 to 15 years in Bangladesh, estimated that 3.5% were OB, 9.5% were OW, and 17.6% were underweight^32^. However recent epidemiological data on OW and OB prevalence in Bangladesh is relatively sparse. Bangladesh is a highly populated emerging nation in South Asia that has experienced dramatic epidemiological and demographic changes over several decades^31^. Even in a resource-poor context, controlling OW and OB is emerging as a major public health challenge, according to these studies^33^. This increase in OW/OB could be attributed to several factors, including fast urbanization, lack of physical exercise, maternal obesity, easy availability of low-cost unhealthy food, and a lack of awareness regarding the risks associated with being OW or OB^30^.

Different criteria have been employed for the assessment of OB/OW, making meaningful comparisons between nations and studies challenging. According to the diagnostic criteria used to analyze the OB/OW status, we observed that the IOTF, WHO, CDC, and EHPA criteria tend to underestimate OB prevalence when compared to IAP criteria, whereas EHPA criteria tend to overestimate OW prevalence among South Asian children and adolescents. However, the results should be interpreted with caution especially those with smaller number of studies. In contrast, a meta-analysis conducted by Mazidi et al. on Asian children and adolescents reported a higher prevalence of OW and OB using the CDC and NCHS criteria^34^.

The majority of studies included were undertaken in metropolitan or urban settings, while few were conducted in rural regions. The prevalence of OB in urban children was 7.3% while the rural prevalence was only 3% which was comparable with the findings of a Chinese study^35^. Perhaps these results can be explained by a combination of rising living standards and consuming an energy-dense diet that is rich in carbohydrates and fat while concurrently exhibiting diminished quantities of essential vitamins and minerals. Moreover, the proliferation of fast food establishments in Asian nations in recent years has resulted in a notable surge in the consumption of unhealthy food options, hence playing a significant role in altering dietary patterns^36^. Urban families also own more televisions and computers than rural families and the increased use of automobiles in urban areas, as opposed to walking or cycling, can also be a contributing cause^37^. Evidence also suggests that lower socioeconomic status (SES) is significantly associated with childhood OB in low-middle-income countries (LMICs)^38^. In LMICs, the availability of nutritious food emerges as a pivotal concern that differentiates those of higher SES from those of lower SES. The affordability of low-calorie food options, such as whole-grain cereals, fruits, and vegetables, may provide a challenge for individuals with limited financial resources. Consequently, this economic constraint may result in the adoption of a diet that is higher in energy density. Furthermore, the process of urbanization and technological advancements in these economies not only impact food consumption but also contribute to a reduction in the physical exertion required for various vocations. As a result, even individuals from lower socioeconomic backgrounds experience a decrease in energy expenditure. Moreover, individuals with lower SES exhibit a heightened vulnerability to the risk of OB due to their limited educational attainment and lower levels of health consciousness^39^.

In our study, gender-based variations were observed. The prevalence of OW and OB was found to be more prevalent in boys than girls. Evidence suggests that girls tend to consume low-calorie-rich foods like fruits and vegetables but boys consume more meat and energy-rich foods. Furthermore, the concerns related to weight will be more in girls, such as eagerness to lose weight and regret of overeating. These factors may potentially contribute to the development of OB in boys. In conjunction with nutritional variables, there are additional sociocultural elements that may exert an influence on the prevalence of OW and OB. Gender disparities are seen in exercising, watching television, and duration of sleep. It is noteworthy that boys of school age exhibit a lower duration of sleep, and spend more time watching television in comparison to their female counterparts^40^.

In recent decades, there has been a general upward trend in OW and OB, and the secular pattern has shifted significantly. Before 2010, the overall prevalence of OB was 4.4%, rising to 5.6% during 2010–2013 and 7.5% during 2014–2018. During 2019–2023, the prevalence of OB increased to 7.6%. A meta-analysis conducted in Bangladesh also found an increasing prevalence of OW and OB over time thereby confirming this trend^30^. If this trend continues, the rising rates of OW and OB may severely compromise the healthcare. Increased demand for health care services would have significant effects on the economic expenses of childhood and adolescent OW or OB-related disorders like congestive heart failure, end-stage renal disease, and many cancers such as endometrial, breast, and gall bladder cancer^41^.

The alarmingly higher rate of OB/OW in South Asia necessitates immediate interventions like educating children, and their family members about the health complications of OB and its associated diseases, educating the community to follow lifestyle changes, such as physical exercise and diet, and implementing national and international monitoring programs to reduce the rate. In South Asian countries, there is no established strategy for controlling OB and OW. The state of Kerala (South India) enacted a 14.5% “fat” tax (tax on pizza, burgers, and other junk food sold in branded stores) in July 2016, which may encourage individuals to make healthier food choices^42^. It is predicted that monitoring and educating for the prevention of diabetes mellitus through National Diabetes Control Programs in several South Asian nations will have a positive impact on OB, however, the efficacy of these approaches has not been thoroughly studied^43^.

### Strength and limitations

This study includes several strengths. The meticulous search technique employed in this study resulted in the inclusion of 152 studies from over 7 countries. This enhances the reliability of the pooled prevalence estimates and provides a more accurate representation of the epidemiology of OB/OW. In addition, our meta-analysis provided a comprehensive assessment of OB/OW in children and adolescents by pooling the prevalence of OB/OW and its subgroups, based on the available evidence.

Several inherent limitations of this study must also be acknowledged. Much fewer studies were conducted in rural areas than in urban areas, limiting our ability to interpret our findings. Different diagnostic criteria were employed across the included studies and hence results should be interpreted with caution. Additionally, the diagnosis of OB and OW were solely based on body mass index (BMI) which results in the exclusion of studies that have reported the prevalence using waist-to-hip ratio and mid-upper arm circumference. All analyses found substantial heterogeneity; however, this is to be expected when compiling data from more than a hundred research that employed diverse criteria and included individuals from various countries and ages.

## Conclusions

The findings indicate that the prevalence of OB and OW was higher in children and adolescents in Bangladesh. Boys residing in urban areas had a far greater prevalence of OB/OW than their rural counterparts. Furthermore, the prevalence of OB was higher with a sample size greater than 1000 and has increased dramatically over the past decade, and they may be considered the most recent trend estimates of OB/OW in children and adolescents. To minimize the prevalence of OW and OB, it is imperative to influence the health habits of children and adolescents through public health prevention methods. It is necessary to conduct additional nationwide, population-based studies on the prevalence of OW and OB in children and adolescents, and these surveys should be representative of the total population. Future surveys should investigate and compensate for these aspects, such as demographic, behavioral, nutritional, social, and economic factors, that influence OB/OW.

### Supplementary Information

Supplementary Information.

## Data Availability

The datasets used and analysed during the current study is available from the corresponding author on reasonable request.
