# Supplementary material for: Trends estimation of obesity prevalence among South Asian young population: a systematic review and meta-analysis
Source: Sci Rep. 2024 Jan 5;14:596. doi: 10.1038/s41598-023-50973-w (PMC10770040; doi:10.1038/s41598-023-50973-w)
Supplement: Supplementary file 1 — Supplementary Information. [file 41598_2023_50973_MOESM1_ESM.docx]

**Supplementary Information**

**Trends estimation of Obesity prevalence among South Asian young population: A Systematic review and meta-analysis**

**Dipika Bansal*^1^, Mohammed Safeer V S^1^,** **Nagita Devi^1^, Chandrasekhar Boya^1^, Karamsetty Dhora Babu^1^, Pinaki Dutta^2^**

1. *Department of Pharmacy Practice, National Institute of Pharmaceutical Education and Research, S.A.S. Nagar, India*
2. *Department of Endocrinology, Postgraduate Institute of Medical Education and Research (PGIMER), Chandigarh, India*

***Corresponding Author:**

Dr Dipika Bansal

Associate Professor

Department of Pharmacy Practice

National Institute of Pharmaceutical Education and Research (NIPER)

S.A.S Nagar,

Punjab-160062, India

Telephone number: +91-9872217542

Email: [dipikabansal079@gmail.com](mailto:dipikabansal079@gmail.com)

**Supplemental Table 1:**  Search strategy to identify studies reporting the prevalence of obesity and overweight

**PubMed**

| **Search No** | **Search terms** | **Hits** |
| --- | --- | --- |
| **#1** | Obese [MeSH terms] OR Obese [All fields] “body-mass index” [All fields] OR Overweight [All fields] OR Obesity [MeSH terms] OR Obesity [All fields] OR” Quetelet Index” [All fields] OR Waist circumference [All fields] OR waist hip ratio [All fields] OR waist to height ratio [All fields] | **939** |
| **#2** | Paediatric [All fields] OR paediatric [All fields] OR new-born [All fields] OR neonat* [All fields] OR infant [All fields] OR adolescen*[All fields] OR adolescent [MeSH Terms] OR adolescent [All fields] OR baby [All fields] OR “pre-school child” [All fields] OR teenage*[All fields] OR toddler [All fields] OR child [All fields] OR child* [All fields] OR children [All fields] OR children [MeSH Terms] | **325** |
| **#3** | “South Asia” [MeSH Terms] OR Diu [All fields]OR Daman[All fields] OR “Andaman Nicobar Islands” [All fields]OR “Jammu Kashmir” [All fields] OR Puducherry[All fields] OR Lakshadweep[All fields] OR Ladakh[All fields] OR Delhi[All fields] OR Chandigarh [All fields]OR West Bengal[All fields] OR Uttarakhand[All fields] OR Uttar Pradesh[All fields] OR Tripura[All fields] OR Telangana[All fields] OR “Tamil Nadu” [All fields] OR Sikkim[All fields] OR Rajasthan[All fields] OR Odisha[All fields] OR Nagaland[All fields] OR Mizoram[All fields] OR Meghalaya[All fields] OR Manipur[All fields] OR Maharashtra[All fields] OR Madhya Pradesh[All fields] OR Kerala [All fields] OR Karnataka[All fields] OR Jharkhand[All fields] OR “Himachal Pradesh” [All fields] OR Haryana[All fields] OR Gujarat[All fields] OR Goa[All fields] OR Chhattisgarh[All fields] OR Bihar[All fields] OR Assam [All fields]OR Arunachal Pradesh [All fields]OR “Andhra Pradesh” [All fields] OR “North India” [All fields] OR “South India” [All fields] OR Indian [All fields] OR India[All fields] OR India[MeSH Terms] OR Maldives[All fields] OR Afghanistan[All fields] OR “Sri Lanka”[All fields] OR Nepal [All fields] OR Pakistan [All fields]OR Bhutan[All fields] OR Bangladesh[All fields] | **996** |
| **#4** | #1 AND #2 AND #3 AND FILTER: Multicenter study OR Observational study | **304** |

**Embase**

| **Search No** | **Search terms** | **Hits** |
| --- | --- | --- |
| **#1** | obese OR (body AND mass AND index) OR overweight OR 'obesity'/exp OR obesity OR quetelet* OR (quetelet AND index) OR (waist AND circumference) OR (waist AND hip AND ratio) OR (waist AND to AND height AND ratio) | 963942 |
| **#2** | paediatric OR pediatric OR newborn OR 'newborn disease'/exp OR 'newborn disease' OR infant OR adolescen* OR adolescent OR baby OR ('pre-school' AND child) OR teenage* OR toddler OR child OR child* OR children | 6331049 |
| **#3** | diu OR daman OR dadras OR 'andaman nicobar islands' OR (andaman AND nicobar AND ('islands'/exp OR islands)) OR (jammu AND kashmir) OR puducherry OR lakshadweep OR ladak OR delhi OR chandigarh OR (west AND bengal) OR uttarakhand OR (uttar AND pradesh) OR tripura OR telangana OR (tamil AND nadu) OR sikkim OR rajasthan OR odisha OR nagaland OR mizoram OR meghalaya OR manipur OR maharashtra OR 'madhya pradesh' OR kerala OR karnataka OR jharkhand | 498256 |
| **#4** | himachal AND pradesh OR haryana OR gujarat OR 'chhattisgarh'/exp OR chhattisgarh OR goa OR bihar OR assam OR (arunachal AND pradesh) OR (andhra AND pradesh) OR 'north indian' OR (south AND india) OR indian OR india* OR maldives OR afghanistan OR (sri AND lanka) OR nepal OR pakistan OR bhutan OR bangladesh OR india | 1658352 |
| **#5** | #3 OR #4 | 1662257 |
| **#6** | #1 AND #2 AND #5 | 11911 |
| **#7** | #6 AND ('Article'/it OR 'Article in Press'/it) | 8135 |
| **#8** | #6 AND ('Article'/it OR 'Article in Press'/it) AND ('cross sectional study'/de OR 'interview'/de OR 'multicenter study'/de OR 'normal human'/de OR 'observational study'/de OR 'prospective study'/de OR 'questionnaire'/de OR 'retrospective study'/de OR 'structured questionnaire'/de) AND ([adolescent]/lim OR [child]/lim OR [infant]/lim OR [newborn]/lim OR [preschool]/lim OR [school]/lim) | 2877 |

**Supplemental Table 2**: Study Characteristics of the included studies (n=152)

| **Author** | **Publication year** | **Country** | **Sample Population** | **Diagnostic Criteria** | **Study centre** | **Study setting** | **Time Frame (months)** | **Age Range (years)** | **Sample Size** | **Overweight N (%)** | **Obesity N (%)** |
| --- | --- | --- | --- | --- | --- | --- | --- | --- | --- | --- | --- |
| Verma et al. [1] | 1994 | India | Punjab | NR | School | Urban | NR | 5 to 15 | 2560 | NR | 131 (5.12) |
| Kapil et al. [2] | 2002 | India | New Delhi | IOTF | School | Urban | NR | 10 to 16 | 870 | 215 (24.7) | 64 (7.4) |
| Ramachandran et al. [3] | 2002 | India | Tamil Nadu | IOTF | School | Urban | NR | 13 to 18 | 4705 | 791 (16.81) | 149 (3.17) |
| Subramanyam et al. [4] | 2003 | India | Tamil Nadu | IOTF | School | Urban | 12 | 10 to 15 | 610 | 59 (9.67) | 38 (6.23) |
| Marwaha et al. [5] | 2006 | India | New Delhi | IOTF | School | Urban | NR | 5 to 17 | 21485 | 2483 (11.56) | 747 (3.48) |
| Sidhu et al. [6] | 2006 | India | Punjab | IOTF | School | Urban | NR | 6 to 11 | 1000 | 133 (13.3) | 61 (6.1) |
| Sharma et al. [7] | 2007 | India | New Delhi | IOTF | School | Urban | 36 | 4 to 17 | 4399 | 987 (22.44) | 282 (6.41) |
| Kaushik et al. [8] | 2007 | India | West Bengal | IOTF | School | Urban | 3 | 6 to 9 | 431 | 76 (17.6) | 22 (5.1) |
| Laxmaiah et al. [9] | 2007 | India | Telangana | IOTF | School | Urban | NR | 12 to 17 | 1208 | 71 (5.9) | 16 (1.3) |
| Manu et al. [10] | 2007 | India | Kerala | CDC | School | Urban | NR | 5 to 16 | 24842 | 1227 (4.94) | 313 (1.26) |
| Sood et al. [11] | 2007 | India | Karnataka | CDC | School | Urban | NR | 9 to 18 | 3970 | 520 (13.1) | 171 (4.31) |
| Aggarwal et al. [12] | 2008 | India | Punjab | Rosner et al. | School | Urban | NR | NR | 1000 | 127 (12.7) | 34 (3.4) |
| Kaur et al. [13] | 2008 | India | New Delhi | IOTF | School | Urban | NR | 5 to 18 | 16595 | 908 (5.47) | 785 (4.73) |
| Kumar et al. [14] | 2008 | India | Karnataka | WHO 2006 | School | Urban | 3 | 2 to 5 | 425 | 19 (4.47) | 6 (1.41) |
| Ramzan et al. [15] | 2008 | Pakistan | Dera Ismail Khan | CDC | School | Rural&Urban | NR | 6 to 11 | 1336 | 118 (8.83) | 75 (5.61) |
| Sultan et al. [16] | 2008 | Bangladesh | Dhaka | WHO 2006 | Hospital | Urban | NR | 0 to 1 | 172 | 44 (25.6) | 24 (14) |
| Bharati et al. [17] | 2008 | India | West Bengal | CDC | School | Urban | NR | 10 to 17 | 2555 | 79 (3.1) | 32 (1.25) |
| Deepa et al. [18] | 2009 | India | Maharashtra | IOTF, CDC, Agarwal et al. | School | Urban | NR | 6 to 17 | 566 | IOTF:53 (9.4), CDC:60 (10.6), Aggarwal:57 (10.1) | IOTF:24 (4.2), CDC:95 (16.8), Aggarwal: 92 (16.3) |
| Ghosh et al. [19] | 2009 | India | West Bengal | WHO 1995 | School | Urban | NR | 9 to 17 | 1153 | 216 (18.8) | NR |
| Jeemon et al. [20] | 2009 | India | Multiple cities | Agarwal et al., IOTF | Community | Urban | NR | 10 to 19 | 3704 | Agarwal:1219 (32.9), IOTF: 852 (23) | NR |
| Aziz et al. [21] | 2009 | Pakistan | Karachi | CDC | School | Urban | 3 | 6 to 17 | 398 | 77 (19.35) | 24 (6) |
| Warraich et al. [22] | 2009 | Pakistan | Karachi | NR | School | Urban | NR | 11 to 17 | 284 | 23 (8.1) | 17 (6) |
| Goyal et al. [23] | 2010 | India | Gujarat | NR | School | Rural&Urban | NR | 12 to 18 | 5664 | 688 (12.15) | 130 (2.3) |
| Bishwalata et al. [24] | 2010 | India | Manipur | WHO 1995, IOTF | School | Rural&Urban | 12 | 12 to 19 | 3356 | WHO: 141 (4.2), IOTF: 149 (4.4) | WHO: 27 (0.8) IOTF: 23 (0.69) |
| Jain et al. [25] | 2010 | India | Uttar Pradesh | EHPA | School | Urban | 6 | 10 to 16 | 2570 | 499 (19.42) | 222 (8.64) |
| Kotain et al. [26] | 2010 | India | Karnataka | IOTF | School | Urban | 4 | 12 to 15 | 900 | 89 (9.9) | 43(4.8) |
| Patnaik et al. [27] | 2010 | India | Orissa | CDC | School | Urban | 2 | 5 to 15 | 468 | 66 (14.1) | 68 (14.53) |
| Premanath et al. [28] | 2010 | India | Karnataka | Agarwal et al. | School | Urban | NR | 5 to 16 | 43152 | 3682 (8.53) | 1448 (3.36) |
| Ramesh et al. [29] | 2010 | India | Kerala | CDC | School | Urban | 6 | 12 to 16 | 1718 | 206 (12) | 108 (6.3) |
| Anwar et al. [30] | 2010 | Pakistan | Lahore | WHO 2007 | School | Urban | 4 | NR | 293 | 64 (21.8) | 35 (11.9) |
| Haq et al. [31] | 2010 | Pakistan | Hazara division | NCHS | School | Rural&Urban | 6 | 5 to 15 | 3200 | NR | 153 (4.78) |
| Khadilkar et al. [32] | 2011 | India | Multiple cities | WHO 2007, IOTF | School | Urban | 8 | 2 to 17 | 20243 | WHO:2247 (11.1), IOTF:3016 (14.9) | WHO:3219 (15.9) IOTF: 951 (4.7) |
| Mihir et al. [33] | 2011 | India | West Bengal | CDC | School | Urban | NR | 9 to 18 | 1568 | 223 (14.2) | 63 (4) |
| Ferdousi et al. [34] | 2011 | Bangladesh | Dhaka | NCHS | School | Urban | 6 | 6 to 10 | 202 | 37 (18.3) | NR |
| Chakraborty et al. [35] | 2011 | India | West Bengal | CDC | School | Urban | 12 | 5 to 18 | 979 | 181 (18.5) | 22 (2.25) |
| Ghosh et al. [36] | 2011 | India | West Bengal | IOTF | School | Rural&Urban | 7 | 8 to 18 | 753 | 73 (9.7) | 42 (5.57) |
| Goyal et al. [37] | 2011 | India | Gujarat | IAP | School | Urban | 10 | 12 to 15 | 1159 | 161 (13.89) | 76 (6.56) |
| Gupta et al. [38] | 2011 | India | New Delhi | IOTF | school | Urban | 36 | 14 to 17 | 8401 | 2075 (24.7) | 903 (10.75) |
| Keerthan et al. [39] | 2011 | India | Karnataka | IAP | School | Rural&Urban | 3 | 12 to 15 | 500 | 13 (2.6) | 15 (3) |
| Mahajan et al. [40] | 2011 | India | Puducherry | CDC | School | Rural&Urban | 5 | 6 to 12 | 2542 | 112 (4.41) | 54 (2.12) |
| Misra et al. [41] | 2011 | India | Multiple cities | IOTF, WHO 2007 | School | Urban | 29 | 8 to 18 | 38296 | IOTF: 5515 (14.4), WHO: 7084 (18.5) | IOTF: 1072 (2.80) WHO: 2030 (5.3) |
| Thakre et al. [42] | 2011 | India | Maharashtra | CDC | School | Urban | 19 | 5 to 18 | 1524 | 137 (8.99) | 84 (5.51) |
| Uma et al. [43] | 2011 | India | Gujarat | CDC, IOTF | School | Urban | NR | 6 to 12 | 1067 | CDC:125(11.7), IOTF: 154 (14.4) | CDC:106 (9.93) IOTF: 63 (5.9) |
| Vohra et al. [44] | 2011 | India | Uttar Pradesh | CDC | School | Urban | NR | NR | 407 | 17 (4.17) | 3 (0.73) |
| Mushtaq et al. [45] | 2011 | Pakistan | Lahore | WHO 2007 | School | Rural&Urban | NR | 5 to 12 | 1860 | 316 (17) | 140 (7.53) |
| Deoke et al. [46] | 2012 | India | Maharashtra | WHO 2007 | School | Urban | NR | 12 to 16 | 565 | 33 (5.8) | 2 (0.4) |
| Sarkar et al. [47] | 2012 | India | Tripura | WHO 2007 | School | Rural&Urban | NR | 8 to 15 | 623 | 23 (3.7) | NR |
| Cherian et al. [48] | 2012 | India | Kerala | CDC | School | Urban | 12 | 6 to 15 | 1634 | 181 (11.1) | 67 (4.1) |
| Maiti et al. [49] | 2013 | India | West Bengal | CDC, IOTF, WHO 2007 | School | Urban | 5 | 10 to 14 | 1375 | IOTF: 103 (7.5), CDC: 105 (7.6), WHO: 146 (10.6) | IOTF: 18 (1.31) CDC:24 (1.75) WHO: 31 (2.25) |
| Wickramasinghe et al. [50] | 2013 | Sri Lanka | Colombo | IOTF | School | Urban | 12 | 5 to 15 | 920 | 90 (9.8) | 32 (3.48) |
| Hashmi et al. [51] | 2013 | Pakistan | Hyderabad | WHO 2007 | School | Urban | 18 | NR | 501 | 98 (19.56) | 58 (11.5) |
| George et al. [52] | 2014 | India | New Delhi | WHO 2007 | School | Urban | 7 | 9 to 18 | 485 | 46 (9.5) | 56 (11.55) |
| Jagadesan et al. [53] | 2014 | India | Tamil Nadu | IOTF&IAP | School | Urban | 36 | 6 to 17 | 18955 | IOTF: 2577(13.6), IAP:3766 (19.9) | IOTF: 645 (3.4) IAP: 1800 (9.5) |
| Kajale et al. [54] | 2014 | India | Multiple cities | IAP | School | Urban | 7 | 6 to 18 | 6380 | 1499 (23) | 619 (10) |
| Tikaram et al. [55] | 2014 | India | Karnataka | IOTF | School | Urban | 1 | 13 to 16 | 300 | 36(12) | 10 (3.3) |
| Parashar et al. [56] | 2014 | India | Uttar Pradesh | NCHS | School | Urban | 4 | 5 to 18 | 4202 | 160 (3.8) | 220 (5.2) |
| Amitha et al. [57] | 2014 | India | Karnataka | Agarwal et al., CDC | School | Urban | 1 | 4 to 16 | 755 | Agarwal: 71(9.4), CDC: 45 (6) | Agarwal: 20 (2.65) CDC: 18 (2.38) |
| Kumaravel et al. [58] | 2014 | India | Tamil Nadu | Agarwal et al., IOTF | School | Rural&Urban | NR | 5 to 18 | 18001 | Agarwal:1710 (9.5), IOTF: 1452 (8.1) | Agarwal: 535 (2.97) IOTF: 468 (2.6) |
| Arnab et al. [59] | 2014 | India | West Bengal | IAP | School | Urban | 39 | 8 to 18 | 1061 | 187 (17.6) | 68 (6.41) |
| Bulbul et al. [60] | 2014 | Bangladesh | Multiple states | WHO 2007 | School | Rural&Urban | 4 | 6 to 15 | 10135 | 977 (9.6) | 359 (3.54) |
| Prasanna et al. [61] | 2015 | India | Karnataka | WHO 2007 | School | Rural | 24 | 10 to 18 | 2963 | 71(2.4) | 42 (1.4) |
| Patnaik et al. [62] | 2015 | India | Odisha | IAP | School | Urban | 6 | 10 to 16 | 1800 | 295 (16) | 206 (11) |
| Koirala et al. [63] | 2015 | Nepal | Lalitpur | WHO 2007 | School | Urban | 7 | 6 to 13 | 986 | 144 (14.6) | 111 (11.26) |
| Goel et al. [64] | 2016 | India | Madhya Pradesh | CDC | Hospital | Urban | 12 | 14 to 17 | 1221 | 112 (9.2) | 69 (5.7) |
| Saikia et al. [65] | 2016 | India | Assam | WHO 2007 | School | Urban | 6 | 10 to 14 | 752 | 169 (22.5) | 73 (9.7) |
| Rajesh et al. [66] | 2016 | India | Gujarat | WHO 2007, IAP, IOTF | School | Urban | 4 | 8 to 18 | 1496 | WHO: 229 (15.3), IAP:285 (19.1), IOTF: 236 (15.8) | WHO: 166(11.1) IAP:210 (14.) IOTF: 77 (5.1) |
| Vishnu et al. [67] | 2016 | India | Pondicherry | IAP | School | Rural&Urban | 7 | 10 to 18 | 2465 | 240 (9.7) | 105 (4.3) |
| Pawar et al. [68] | 2016 | India | Maharashtra | IOTF, IAP | School | Urban | 2 | 9 to 15 | 1828 | IOTF: 207 (11.3), IAP: 320 (17.5) | IOTF: 62 (3.4) IAP: 143 (7.8) |
| Aluckal et al. [69] | 2016 | India | Karnataka | CDC | School | Rural | NR | 2 to 6 | 433 | 26 (6) | NR |
| Bhargava et al. [70] | 2016 | India | Uttarakhand | WHO 2007 | School | Rural&Urban | 12 | 6 to 17 | 1266 | 197 (15.6) | 68 (5.4) |
| Hussain et al. [71] | 2016 | India | Karnataka | NR | School | Urban | NR | 10 to 15 | 1642 | 217 (13.2) | 112 (6.8) |
| Babitha et al. [72] | 2016 | India | Tamil Nadu | IAP | School | Rural&Urban | NR | 6 to 12 | 2519 | 423 (16.8) | 234 (9.3) |
| Nawab et al. [73] | 2016 | India | Uttar Pradesh | WHO 2007 | School | Urban | 12 | 10 to 16 | 660 | 65 (9.8) | 32 (4.8) |
| Sultana et al. [74] | 2016 | Bangladesh | Dhaka | WHO 2007 | School | Urban | 6 | 6 to 13 | 1200 | 159 (13.3) | 213 (17.75) |
| Bharati et al. [75] | 2017 | India | West Bengal | CDC | School | Urban | NR | 6 to 10 | 5216 | 532 (10.2) | 1158 (22.2) |
| Mishra et al. [76] | 2017 | India | Orissa | IOTF | School | Urban | 12 | 10 to 12 | 300 | 19 (6.3) | 10 (3.3) |
| Ganie et al. [77] | 2017 | India | Jammu and Kashmir | CDC | School | Rural&Urban | 22 | 6 to 18 | 2024 | 95 (4.7) | 94 (4.6) |
| Faizi et al. [78] | 2017 | India | Uttar Pradesh | WHO 2007 | School | Urban | 9 | 13 to 16 | 1456 | 174 (12) | 33 (2.3) |
| Cramer et al. [79] | 2018 | India | New Delhi | WHO 2007 | School | Urban | 24 | 10 to 16 | 551 | 163 (29.6) | 162 (29.4) |
| Jagannath et al. [80] | 2018 | India | Maharashtra | NR | School | Rural | 5 | 11 to 16 | 207 | 25 (12.1) | 18 (8.7) |
| Rebecca et al. [81] | 2018 | India | Karnataka | WHO 2007 | School | Urban | 60 | 6 to 15 | 9702 | 1261 (13) | 485 (5) |
| Sonali et al. [82] | 2018 | India | West Bengal | WHO 2007 | School | Rural&Urban | NR | 6 to 12 | 1227 | 155 (12.6) | 46 (3.75) |
| Mansoori et al. [83] | 2018 | Pakistan | Karachi | CDC | School | Urban | 9 | 11 to 15 | 887 | 169 (19.1) | 96 (10.82) |
| Sajon et al. [84] | 2018 | Bangladesh | Khulna | NR | School | Urban | 7 | 7 to 18 | 300 | 75 (25) | 49 (16.33) |
| Sultana et al. [85] | 2019 | Bangladesh | Dhaka | WHO 2007, IOTF, CDC | School | Urban | 12 | 6 to 12 | 1768 | WHO:192 (10.9), IOTF:112 (6.3), CDC: 177 (10) | WHO:74 (4.19) IOTF: 84 (4.75) CDC: 93 (5.26) |
| Mohan et al. [86] | 2019 | India | Punjab | IAP | School | Rural&Urban | 17 | 11 to 17 | 1959 | 187 (9.5) | 125 (6.4) |
| Chandra et al. [87] | 2019 | India | Telangana | IAP&CDC | School | Urban | NR | 9 to 15 | 544 | IAP: 195 (35.8), CDC: 142 (26.1) | IAP: 134 (24.6) CDC: 84 (15.4) |
| Dolkar et al. [88] | 2019 | India | Sikkim | WHO 2007 | School | Rural&Urban | 22 | 10 to 19 | 616 | 79 (12.8) | 17 (2.8) |
| Dabade et al. [89] | 2019 | India | Maharashtra | IAP | School | Rural&Urban | 3 | 12 to 16 | 360 | 33 (9.2) | 9 (2.5) |
| Khan et al. [90] | 2019 | Pakistan | Islamabad, Peshawar | WHO 2007 | Hospital | Urban | NR | 2 to 16 | 3500 | 37 (1.1) | 46 (1.31) |
| Karki et al. [91] | 2019 | Nepal | Lalitpur | WHO 2007 | School | Urban | 6 | 6 to 13 | 575 | 107 (18.6) | 41 (7.13) |
| Manandhar et al. [92] | 2019 | Nepal | Kathmandu | NR | School | Urban | 1.5 | 6 to 13 | 440 | 58 (13.2) | 30 (6.82) |
| Norbu et al. [93] | 2019 | Bhutan | Pemaghatsel | CDC | School | Rural | NR | 13 to 17 | 392 | 28 (7.1) | 6 (1.53) |
| Daga et al. [94] | 2020 | India | Maharashtra | NR | School | Rural | NR | 3 to 12 | 1444 | 36 (2.5) | 21 (1.5) |
| Kaur et al. [95] | 2020 | India | Rajasthan | CDC | Community | Rural&Urban | NR | 10 to 17 | 240 | 7 (2.9) | 5 (2.1) |
| Singh et al. [96] | 2020 | India | New Delhi | Agarwal et al., IAP&WHO 2007 | School | Urban | 1 | 8 to 15 | 1237 | IAP:237 (19.2), Aggarwal: 244 (19.7), WHO: 224 (18.1) | IAP:199 (16.1) Aggarwal: 150 (12.1) WHO: 136 (11) |
| Kavi et al. [97] | 2020 | India | Karnataka | IOTF | School | Urban | NR | 12 to 18 | 613 | 31(5.1) | 34 (5.5) |
| Viswanathan et al. [98] | 2020 | India | Maharashtra | WHO 2007, IAP | School | Rural | 8 | 12 to 15 | 300 | WHO: 7 (2.3), IAP: 27 (9) | WHO: 15 (5) IAP: 15 (5) |
| Bhuvaneswari et al. [99] | 2020 | India | Tamil Nadu | CDC | School | Urban | NR | 13 to 18 | 514 | 127(24.7) | 387 (75.3) |
| Solanki et al. [100] | 2020 | India | Chandigarh | CDC | School | Urban | 18 | 10 to 18 | 10037 | 930 (9.3) | 487 (4.9) |
| Naskar et al. [101] | 2020 | India | West Bengal | WHO 2007 | School | Urban | NR | 10 to 19 | 570 | 72 (12.6) | 20 (3.5) |
| Hassan et al. [102] | 2020 | Bangladesh | Noakhali | IOTF, CDC, WHO 2007 | School | Urban | 12 | 14 to 19 | 825 | IOTF:86 (10.4), CDC: 81 (9.8), WHO: 88 (10.7) | IOTF: 11 (1.33) CDC:23 (2.79) WHO: 41 (4.97) |
| Dhirendra et al. [103] | 2020 | India | Chandigarh | IAP | School | Urban | NR | 10 to 14 | 1030 | 102 (9.9) | 144 (14) |
| Tariq et al. [104] | 2021 | Pakistan | Faisalabad | WHO 2007 | School | Urban | 4 | 13 to 19 | 226 | 28 (12.4) | 19 (8.41) |
| Sathiadas et al. [105] | 2021 | Sri Lanka | Jaffna | WHO 2007 | School | Rural&Urban | 10 | 6 to 16 | 1012 | 112 (11.1) | 64 (6.32) |
| Bhat et al. [106] | 2021 | India | Rajasthan | CDC | School | Urban | 6 | 13 to 17 | 500 | 57 (11.4) | NR |
| Bodhare et al. [107] | 2021 | India | Tamil Nadu | WHO 2007 | School | Urban | NR | 10 to 14 | 544 | 49 (9) | 5 (0.92) |
| Das et al. [108] | 2021 | India | Orissa | IAP | School | Urban | 12 | 6 to 16 | 1930 | 383 (19.8) | 162 (8.4) |
| Malarvizhi et al. [109] | 2021 | India | Tamil Nadu | NR | School | Urban | 1 | 11 to 13 | 100 | 19 (19) | 27 (27) |
| Mangalam et al. [110] | 2021 | India | Bihar | IOTF | School | Urban | 9 | 8 to 15 | 660 | 120 (18.18) | 72 (10.9) |
| Mathew et al. [111] | 2021 | India | Karnataka | WHO 2007 | School | Urban | 3 | 13 to 15 | 360 | 35 (9.7) | 30 (8.3) |
| Moudgil et al. [112] | 2021 | India | Chandigarh | IAP | Hospital | Urban | NR | 6 to 17 | 154 | 15 (9.7) | 8 (5.2) |
| Paladugu et al. [113] | 2021 | India | Andhra Pradesh | WHO 2007 | Community | Urban | 6 | 6 to 12 | 346 | 36 (10.4) | 62 (17.9) |
| Qaisar et al. [114] | 2021 | Pakistan | Punjab | CDC, IOTF, WHO 2007 | School | Urban | 1 | 8 to 16 | 10050 | CDC:533 (5.3), IOTF: 734 (7.3), WHO: 794 (7.9) | CDC: 191(1.9) IOTF: 151 (1.5) WHO: 221 (2.2) |
| Rawal et al. [115] | 2021 | India | New Delhi | WHO 2007 | School | Urban | NR | 11 to 12 | 1564 | 307(19.6) | 185 (11.8) |
| Saeedullah et al. [116] | 2021 | Pakistan | Peshawar | WHO 2007 | Community | Rural | 1 | 10 to 19 | 206 | 24 (11.7) | 6 (2.9) |
| Salvi et al. [117] | 2021 | India | Multiple states | IAP | School | Urban | NR | 13 to 17 | 3157 | 1168 (37) | 606 (19.2) |
| Sarna et al. [118] | 2021 | India | Multiple states | WHO 2007 | Community | Rural&Urban | NR | 5 to 19 | 67741 | NR | 745 (1.1) |
| Siva et al. [119] | 2021 | India | Tamil Nadu | WHO 2007 | School | Rural | NR | 6 to 12 | 280 | 200 (71.4) | NR |
| Syamkumar et al. [120] | 2021 | India | Karnataka | CDC | School | Urban | NR | 13 to 15 | 660 | 85 (12.9) | NR |
| Thapa et al. [121] | 2021 | Nepal | Kathmandu | WHO 2007 | School | Urban | 13 | 5 to 18 | 379 | 31 (8.2) | 7 (1.8) |
| Anam et al.[122] | 2022 | Bangladesh | Ghazipur | WHO 2007 | School | Rural & Urban | NR | 13 to 17 | 1044 | 187 (17.9) | 74 (7.1) |
| Arushi et al.[123] | 2022 | India | Rajasthan | WHO 2007 | School | Rural & Urban | NR | 10 to 18 | 1620 | 229 (14.1) | 76 (4.7) |
| Asghar et al.[124] | 2022 | India | Arunachal Pradesh | WHO 1995 | Community | Rural | NR | 5 to 18 | 452 | 80 (17.7) | NR |
| Banerjee et al.[125] | 2022 | India | Jammu & Kashmir | WHO 2007 | School | Rural & Urban | NR | 6 to 18 | 1675 | 147 (8.8) | 78 (4.7) |
| Bekhwani et al.[126] | 2022 | Pakistan | Karachi | NR | Hospital | Rural & Urban | NR | 5 to 16 | 184 | 25 (13.6) | 80 (43.5) |
| Bhusal et al.[127] | 2022 | Nepal | Kapilvastu | WHO 2006 | Community | Rural & Urban | NR | 0 to 5 | 336 | 3 (0.9) | NR |
| Cazzaniga et al.[128] | 2022 | Maldives | Maghoodoo Island | CDC | Community | Rural & Urban | NR | NR | 145 | 20 (13.8) | NR |
| Chanchala et al.[129] | 2022 | India | Karnataka | IAP | School | Rural & Urban | 10 | 12 | 1602 | 113 (7.1) | 88 (5.5) |
| Dabas et al.[130] | 2022 | India | New Delhi | IOTF, IAP | School | Rural & Urban | NR | 10 to 18 | 8417 | 664 (7.9), 1234 (14.7) | 230 (2.7), 716 (8.5) |
| Ghayasuddin et al.[131] | 2022 | Pakistan | Peshawar | NR | School | Rural & Urban | 9 | 6 to 15 | 300 | 25 (8.3) | 21 (7) |
| Jubayer et al.[132] | 2022 | Bangladesh | St. Martins Island | WHO 2006 | Community | Rural & Urban | 2 | 0 to 15 | 256 | 19 (7.4) | NR |
| Mogra et al.[133] | 2022 | India | Madhya Pradesh | WHO 2007 | School | Urban | NR | 12 to 15 | 2000 | 223 (11.2) | 176 (8.8) |
| Pradhan et al.[134] | 2022 | India | Orissa | NR | School | Rural & Urban | 2 | 13 to 18 | 180 | 17 (9.4) | 9 (5) |
| Tanveer et al.[135] | 2022 | Pakistan | Multiple cities | CDC | School | Rural & Urban | NR | 9 to 17 | 3551 | 205 (5.8) | 191 (5.4) |
| Thilagar et al.[136] | 2022 | India | New Delhi | WHO 2007 | School | Urban | 18 | NR | 244 | 29 (11.9) | 7 (2.9) |
| Uprety et al.[137] | 2022 | Nepal | Bhaktapur | WHO 2006 | Hospital | Urban | 5 | 0 to 2 | 305 | 7 (2.3) | NR |
| Yadawa et al.[138] | 2022 | India | Bihar | CDC | School | Rural & Urban | 12 | 14 to 18 | 492 | 36 (7.3) | 17 (3.5) |
| Akthar et al.[139] | 2023 | Pakistan | Karachi | CDC | School | Urban | 2 | 11 to 16 | 364 | 20 (5.5) | 8 (2.2) |
| Ashraf et al.[140] | 2023 | Pakistan | Rawalpindi | WHO 2007 | Hospital | Rural & Urban | 3 | NR | 519 | 48 (9.2) | 31 (6) |
| Bamania et al.[141] | 2023 | India | Gujarat | IAP | School | Rural & Urban | 1 | 10 to 15 | 101 | 15 (14.9) | NR |
| Bhattad et al.[142] | 2023 | India | Maharashtra | NR | School | Rural & Urban | 6 | 12 to 16 | 500 | 49 (9.8) | 25 (5) |
| Dahal et al.[143] | 2023 | Nepal | Panauti | NR | School | Rural & Urban | NR | 0 to 5 | 226 | 50 (22.1) | NR |
| Hamann et al.[144] | 2023 | Nepal | Pokhara | WHO 2007 | School | Rural & Urban | NR | 9 to17 | 868 | 108 (12.4) | 37 (4.3) |
| Herkar et al.[145] | 2023 | India | Maharashtra | NR | School | Rural & Urban | NR | 12 to 15 | 600 | 104 (17.3) | 69 (11.5) |
| Hossain et al.[146] | 2023 | Bangladesh | Jamalpur | CDC | School | Urban | 2 | 4 to 7 | 585 | 34 (5.8) | 48 (8.2) |
| Khatri et al.[147] | 2023 | Nepal | Makwanpur | WHO 2007 | School | Urban | NR | 10 to 19 | 279 | 26 (9.3) | NR |
| Manna et al.[148] | 2023 | India | West Bengal | WHO 2007 | Hospital | Rural & Urban | 3 | 10 to 19 | 178 | 40 (22.5) | NR |
| Rajnikanth et al.[149] | 2023 | India | Tamil Nadu | IAP | School | Urban | NR | 12 to 16 | 500 | 113 (22.6) | 91 (18.2) |
| Santra et al.[150] | 2023 | India | New Delhi | WHO 2007 | Hospital | Urban | 3 | 10 to 19 | 386 | 32 (8.3) | 4 (1) |
| Sivabalan et al.[151] | 2023 | India | Tamil Nadu | NR | Hospital | Rural & Urban | 12 | 12 to 16 | 500 | 109 | NR |
| Sonar et al.[152] | 2023 | India | Maharashtra | NR | Hospital | Rural & Urban | 12 | 6 to 12 | 200 | NR | 60 (30) |

*WHO: World Health Organisation; IOTF: International Obesity Task Force; IAP: Indian Academy of Paediatrics; CDC: Centre for Disease Control and Prevention; NCHS: National Centre for Health Statistics; EHPA: Eliz health path for adolescents and adults (EHPA); NR: Not reported*

**Supplemental Table 3**: Risk of Bias of included studies (n=152)

| **Sl.No.** | **Study Id (Year)** | **Was the sample frame appropriate to address the target population?** | **Were study participants sampled in an appropriate way?** | **Was the sample size adequate?** | **Were the study subjects and the setting described in detail?** | **Was the data analysis conducted with sufficient coverage of the identified sample** | **Were valid methods used for the identification of the condition?** | **Was the condition measured in a standard, reliable way for all participants?** | **Was there appropriate statistical analysis?** | **Was the response rate adequate, and if not, was the low response rate managed appropriately?** | **Score** | **Percentage** |
| --- | --- | --- | --- | --- | --- | --- | --- | --- | --- | --- | --- | --- |
| 1 | Verma et al. (1994) | Yes | No | No | Yes | Yes | No | Yes | Yes | Yes | 6 | 66.67 |
| 2 | Kapil et al. (2002) | Yes | Yes | Yes | Yes | Yes | Yes | Yes | Yes | Yes | 9 | 100.00 |
| 3 | Ramachandran et al. (2002) | Yes | No | No | Yes | Yes | Yes | Yes | Yes | Yes | 7 | 77.78 |
| 4 | Subrahmanyam et al. (2003) | Yes | No | No | Yes | Yes | Yes | Yes | Yes | Yes | 7 | 77.78 |
| 5 | Marwaha et al. (2006) | Yes | No | No | Yes | Yes | Yes | Yes | Yes | Yes | 7 | 77.78 |
| 6 | Sidhu et al. (2006) | Yes | No | No | No | Yes | Yes | Yes | Yes | Yes | 6 | 66.67 |
| 7 | Sharma et al. (2007) | Yes | No | No | Yes | Yes | Yes | Yes | Yes | Yes | 7 | 77.78 |
| 8 | Kaushik et al. (2007) | Yes | No | No | Yes | Yes | Yes | Yes | Yes | Yes | 7 | 77.78 |
| 9 | Laxmaiah et al. (2007) | Yes | Yes | Yes | Yes | Yes | Yes | Yes | Yes | Yes | 9 | 100.00 |
| 10 | Manu et al. (2007) | Yes | Yes | Yes | Yes | Yes | Yes | Yes | Yes | Yes | 9 | 100.00 |
| 11 | Sood et al. (2007) | Yes | Yes | No | Yes | Yes | Yes | Yes | Yes | Yes | 8 | 88.89 |
| 12 | Aggarwal et al. (2008) | Yes | Yes | No | No | Yes | Unclear | Yes | Yes | Yes | 6 | 66.67 |
| 13 | Kaur et al. (2008) | Yes | No | Yes | Yes | Yes | Yes | Yes | Yes | Yes | 8 | 88.89 |
| 14 | Kumar et al. (2008) | Yes | Yes | Yes | Yes | Yes | Yes | Yes | No | Yes | 8 | 88.89 |
| 15 | Ramzan et al. (2008) | Yes | No | No | Yes | Yes | Yes | Yes | Yes | Yes | 7 | 77.78 |
| 16 | Sultan et al. (2008) | Yes | No | No | Yes | Yes | Yes | Yes | Yes | Yes | 7 | 77.78 |
| 17 | Bharati et al. (2008) | Yes | Yes | Yes | Yes | Yes | Yes | Yes | Yes | Yes | 9 | 100.00 |
| 18 | Deepa et al. (2009) | Yes | Yes | Yes | Yes | Yes | Yes | Yes | Yes | Unclear | 8 | 88.89 |
| 19 | Ghosh et al. (2009) | Yes | Yes | Yes | Yes | Yes | Yes | Unclear | Yes | Unclear | 7 | 77.78 |
| 20 | Jeemon et al. (2009) | Yes | Yes | No | Yes | Yes | Yes | Yes | Yes | Yes | 8 | 88.89 |
| 21 | Aziz et al. (2009) | Yes | Yes | No | Yes | Yes | Yes | Yes | Yes | Yes | 8 | 88.89 |
| 22 | Warraich et al. (2009) | Yes | Yes | Yes | Yes | Yes | Yes | Yes | Yes | Yes | 9 | 100.00 |
| 23 | Goyal et al. (2010) | Yes | No | No | Yes | Yes | No | Yes | Yes | Yes | 6 | 66.67 |
| 24 | Bishwalata et al. (2010) | Yes | Yes | Yes | Yes | Yes | Yes | Yes | Yes | Yes | 9 | 100.00 |
| 25 | Jain et al. (2010) | Yes | Yes | Yes | Yes | Yes | Yes | Yes | Yes | Yes | 9 | 100.00 |
| 26 | Kotain et al. (2010) | Yes | Yes | Yes | Yes | Yes | Yes | Yes | Yes | Yes | 9 | 100.00 |
| 27 | Patnaik et al. (2010) | Yes | Yes | No | Yes | Yes | Yes | Yes | Yes | Yes | 8 | 88.89 |
| 28 | Premanath et al. (2010) | Yes | Yes | Yes | Yes | Yes | Yes | Yes | Yes | Yes | 9 | 100.00 |
| 29 | Ramesh et al. (2010) | Yes | Yes | Yes | Yes | Yes | Yes | Yes | Yes | Yes | 9 | 100.00 |
| 30 | Anwar et al. (2010) | Yes | Yes | No | Yes | Yes | Yes | Yes | Yes | Yes | 8 | 88.89 |
| 31 | Haq et al. (2010) | Yes | No | No | Yes | Yes | No | Yes | Yes | Yes | 6 | 66.67 |
| 32 | Khadilkar et al. (2011) | Yes | Yes | Yes | Yes | Yes | Yes | Yes | Yes | Yes | 9 | 100.00 |
| 33 | Mihir et al. (2011) | Yes | Yes | Yes | Yes | Yes | Yes | Yes | Yes | Unclear | 8 | 88.89 |
| 34 | Ferdousi et al. (2011) | Yes | Yes | Yes | Yes | Yes | Yes | Yes | Yes | Yes | 9 | 100.00 |
| 35 | Chakraborty et al. (2011) | Yes | Yes | Yes | Yes | Yes | Yes | Yes | Yes | Yes | 9 | 100.00 |
| 36 | Ghosh et al. (2011) | Yes | Yes | Yes | Yes | Yes | No | Unclear | Yes | Yes | 7 | 77.78 |
| 37 | Goyal et al. (2011) | Yes | Yes | Yes | Yes | Yes | Yes | Yes | Yes | Yes | 9 | 100.00 |
| 38 | Gupta et al. (2011) | Yes | Yes | Yes | Yes | Yes | Yes | Yes | Yes | Yes | 9 | 100.00 |
| 39 | Keerthan et al. (2011) | Yes | No | No | Yes | Yes | Yes | Yes | Yes | Yes | 7 | 77.78 |
| 40 | Mahajan et al. (2011) | Yes | Yes | Yes | Yes | Yes | Yes | Yes | Yes | Yes | 9 | 100.00 |
| 41 | Misra et al. (2011) | Yes | Yes | Yes | Yes | Yes | Yes | Yes | Yes | Yes | 9 | 100.00 |
| 42 | Thakre et al. (2011) | Yes | Yes | Yes | Yes | Yes | Yes | Yes | Yes | Yes | 9 | 100.00 |
| 43 | Uma et al. (2011) | Yes | No | Yes | Yes | Yes | Yes | Yes | Yes | Yes | 8 | 88.89 |
| 44 | Vohra et al. (2011) | Yes | Yes | Yes | Yes | Yes | Yes | Yes | Yes | Yes | 9 | 100.00 |
| 45 | Mushtaq et al. (2011) | Yes | Yes | Yes | Yes | Yes | Yes | Yes | Yes | Yes | 9 | 100.00 |
| 46 | Deoke et al. (2012) | Yes | Yes | Yes | Yes | Yes | Yes | Yes | Yes | Yes | 9 | 100.00 |
| 47 | Sarkar et al. (2012) | Yes | Yes | Yes | Yes | Yes | Yes | Unclear | Yes | Unclear | 7 | 77.78 |
| 48 | Cherian et al. (2012) | Yes | No | No | Yes | Yes | Yes | Yes | Yes | Yes | 7 | 77.78 |
| 49 | Maiti et al. (2013) | Yes | Yes | Yes | Yes | Yes | Yes | Unclear | Yes | Yes | 8 | 88.89 |
| 50 | Wickramasinghe et al. (2013) | Yes | Yes | Yes | Yes | Yes | Yes | Yes | Yes | Yes | 9 | 100.00 |
| 51 | Hasmi et al. (2013) | Yes | Yes | Yes | Yes | Yes | Yes | Yes | Yes | Yes | 9 | 100.00 |
| 52 | George et al. (2014) | Yes | No | Yes | Yes | Yes | Yes | Unclear | Yes | Unclear | 6 | 66.67 |
| 53 | Jagadesan et al. (2014) | Yes | Yes | Yes | Yes | Yes | Yes | Yes | Yes | Yes | 9 | 100 |
| 54 | Kajale et al. (2014) | Yes | Yes | Yes | Yes | Yes | Yes | Yes | Yes | Unclear | 8 | 88.89 |
| 55 | Tikaram et al. (2014) | Yes | Yes | Yes | Yes | Yes | Yes | Unclear | Yes | Unclear | 7 | 77.78 |
| 56 | Parashar et.al. (2014) | Yes | Yes | Yes | Yes | Yes | Yes | Unclear | Yes | Unclear | 7 | 77.78 |
| 57 | Amitha et al. (2014) | Yes | Yes | Yes | Yes | Yes | Yes | Yes | Yes | Yes | 9 | 100.00 |
| 58 | Kumaravel et al. (2014) | Yes | Yes | Yes | Yes | Yes | Yes | Yes | Yes | Unclear | 8 | 88.89 |
| 59 | Arnab et al. (2014) | Yes | Yes | Yes | Yes | Yes | Yes | Unclear | yes | Yes | 8 | 88.89 |
| 60 | Bulbul et al. (2014) | Yes | Yes | Yes | Yes | Yes | Yes | Unclear | Yes | Unclear | 7 | 77.78 |
| 61 | Prasanna et al. (2015) | Yes | Yes | Yes | Yes | Yes | Yes | Yes | Yes | Yes | 9 | 100.00 |
| 62 | Patnaik et al. (2015) | Yes | Yes | Yes | Yes | Yes | Yes | Unclear | Yes | Unclear | 7 | 77.78 |
| 63 | Koirala et.al. (2015) | Yes | No | Yes | Yes | Yes | Yes | Yes | Yes | Yes | 8 | 88.89 |
| 64 | Goel et al. (2016) | Yes | Yes | Yes | Yes | Yes | Yes | Unclear | Yes | Unclear | 7 | 77.78 |
| 65 | Saikia et al. (2016) | Yes | Yes | Yes | Yes | Yes | Yes | Yes | Yes | Yes | 9 | 100.00 |
| 66 | Rajesh et al. (2016) | Yes | Yes | Yes | Yes | Yes | Yes | Yes | Yes | Unclear | 8 | 88.89 |
| 67 | Vishnu et al. (2016) | Yes | Yes | Yes | Yes | Yes | Yes | Unclear | Yes | Unclear | 7 | 77.78 |
| 68 | Pawar et al. (2016) | Yes | No | Yes | Yes | Yes | Yes | Unclear | Yes | Unclear | 6 | 66.67 |
| 69 | Aluckal et al. (2016) | Yes | Yes | Yes | Yes | Yes | Yes | Unclear | Yes | Unclear | 7 | 77.78 |
| 70 | Bhargava et al. (2016) | Yes | Yes | Yes | Yes | Yes | Yes | Unclear | Yes | Unclear | 7 | 77.78 |
| 71 | Hussain et al. (2016) | Yes | Yes | Yes | Yes | Yes | Yes | Yes | Yes | Unclear | 8 | 88.89 |
| 72 | Babitha et al. (2016) | Yes | Yes | Yes | Yes | Yes | Yes | Unclear | Yes | Unclear | 7 | 77.78 |
| 73 | Nawab et al. (2016) | Yes | Yes | Yes | Yes | Yes | Yes | Yes | Yes | Yes | 9 | 100.00 |
| 74 | Sultana et al. (2016) | Yes | Yes | Yes | Yes | Yes | Yes | Yes | Yes | Unclear | 8 | 88.89 |
| 75 | Bharati et al. (2017) | Yes | Yes | Yes | Yes | Yes | Yes | Unclear | Yes | Unclear | 7 | 77.78 |
| 76 | Mishra et al. (2017) | Yes | Yes | Yes | Yes | Yes | Yes | Unclear | Yes | Unclear | 7 | 77.78 |
| 77 | Ganie et al. (2017) | Yes | Yes | Yes | Yes | Yes | Yes | Yes | Yes | Unclear | 8 | 88.89 |
| 78 | Faizi et al. (2017) | Yes | Unclear | Yes | Yes | Yes | Yes | Unclear | Yes | Unclear | 6 | 66.67 |
| 79 | Cramer et al. (2018) | Yes | Yes | Yes | Yes | Yes | Yes | Yes | Yes | Yes | 9 | 100.00 |
| 80 | Jagannath et al. (2018) | Yes | Yes | Yes | Yes | Yes | Yes | Unclear | Yes | Unclear | 7 | 77.78 |
| 81 | Rebecca et al (2018) | Yes | Yes | Yes | Yes | Yes | Yes | Unclear | Yes | Yes | 8 | 88.89 |
| 82 | Sonali et al. (2018) | Yes | Yes | Yes | Yes | Yes | Yes | Unclear | Yes | Unclear | 7 | 77.78 |
| 83 | Mansoori et al. (2018) | Yes | Yes | Yes | Yes | Yes | Yes | Yes | Yes | Yes | 9 | 100.00 |
| 84 | Sajon et al. (2018) | Yes | Yes | Yes | Yes | Yes | Yes | Unclear | Yes | Yes | 8 | 88.89 |
| 85 | Sultana et al. (2019) | Yes | Yes | Yes | Yes | Yes | Yes | Unclear | Yes | Unclear | 7 | 77.78 |
| 86 | Mohan et al. (2019) | Yes | Yes | Yes | Yes | Yes | Yes | Yes | Yes | Unclear | 8 | 88.89 |
| 87 | Chandra et al. (2019) | Yes | Unclear | Yes | Yes | Yes | Yes | Unclear | No | Unclear | 5 | 55.56 |
| 88 | Dolkar et al. (2019) | Yes | Yes | Yes | Yes | Yes | Yes | Yes | Yes | Unclear | 8 | 88.89 |
| 89 | Dabade et al. (2019) | Yes | Yes | Yes | Yes | Yes | Yes | Unclear | Yes | Unclear | 7 | 77.78 |
| 90 | Khan et al. (2019) | Yes | Yes | Yes | Yes | Yes | Yes | Unclear | Yes | Unclear | 7 | 77.78 |
| 91 | Karki et al. (2019) | Yes | Yes | Yes | Yes | Yes | Yes | Yes | Yes | Yes | 9 | 100.00 |
| 92 | Manandhar et al. (2019) | Yes | Yes | Yes | Yes | Yes | Yes | Yes | Yes | Yes | 9 | 100.00 |
| 93 | Norbu et al. (2019) | Yes | No | No | Yes | Yes | Yes | Yes | Yes | Yes | 7 | 77.78 |
| 94 | Daga et al. (2020) | Yes | Yes | Yes | Yes | Yes | Yes | Unclear | Yes | Unclear | 7 | 77.78 |
| 95 | Kaur et al. (2020) | Yes | Yes | Yes | Yes | Yes | Yes | Yes | Yes | Unclear | 8 | 88.89 |
| 96 | Singh et al. (2020) | Yes | Yes | Yes | Yes | Yes | Yes | Yes | Yes | Yes | 9 | 100.00 |
| 97 | Kavi et al. (2020) | Yes | Yes | Yes | Yes | Yes | Yes | Unclear | Yes | Unclear | 7 | 77.78 |
| 98 | Viswanathan et al. (2020) | Yes | Yes | Yes | Yes | Yes | Yes | Unclear | Yes | Unclear | 7 | 77.78 |
| 99 | Bhuvaneswari et al. (2020) | Yes | Yes | Yes | Yes | Yes | Yes | Unclear | Yes | Yes | 8 | 88.89 |
| 100 | Solanki et al. (2020) | Yes | Yes | Yes | Yes | Yes | Yes | Yes | Yes | Yes | 9 | 100.00 |
| 101 | Naskar et al. (2020) | Yes | Yes | Yes | Yes | Yes | Yes | Yes | Yes | Yes | 9 | 100.00 |
| 102 | Hassan et al. (2020) | Yes | Yes | Yes | Yes | Yes | Yes | Unclear | Yes | Unclear | 7 | 77.78 |
| 103 | Dhirendra et al. (2020) | Yes | Yes | Yes | Yes | Yes | Yes | Unclear | Yes | Unclear | 7 | 77.78 |
| 104 | Tariq et al. (2020) | Yes | Yes | Yes | Yes | Yes | Yes | Unclear | Yes | Unclear | 7 | 77.78 |
| 105 | Sathiadas et al. (2021) | Yes | Yes | Yes | Yes | Yes | Yes | Yes | Yes | Yes | 9 | 100.00 |
| 106 | Bhat et al. (2021) | Yes | No | No | Yes | Yes | Yes | Yes | Yes | Yes | 7 | 77.78 |
| 107 | Bodhare et al. (2021) | Yes | Yes | Yes | Yes | Yes | Yes | Yes | Yes | Yes | 9 | 100.00 |
| 108 | Das et al. (2021) | Yes | Yes | Yes | Yes | Yes | Yes | Yes | Yes | Yes | 9 | 100.00 |
| 109 | Malarvizhi et al. (2021) | Yes | No | No | Yes | Yes | No | Yes | Yes | Yes | 6 | 66.67 |
| 110 | Mangalam et al. (2021) | Yes | No | No | Yes | Yes | Yes | Yes | Yes | Yes | 7 | 77.78 |
| 111 | Mathew et al. (2021) | Yes | Yes | Yes | Yes | Yes | Yes | Yes | Yes | Yes | 9 | 100.00 |
| 112 | Moudgil et al. (2021) | Yes | No | No | No | Yes | Yes | Yes | Yes | Yes | 6 | 66.67 |
| 113 | Paladugu et al. (2021) | Yes | No | No | Yes | Yes | Yes | Yes | Yes | Yes | 7 | 77.78 |
| 114 | Qaisar et al. (2021) | Yes | Yes | No | Yes | Yes | Yes | Yes | Yes | Yes | 8 | 88.89 |
| 115 | Rawal et al. (2021) | Yes | Yes | No | Yes | Yes | Yes | Yes | Yes | Yes | 8 | 88.89 |
| 116 | Saeedullah et al. (2021) | Yes | Yes | Yes | Yes | Yes | Yes | Yes | Yes | Yes | 9 | 100.00 |
| 117 | Salvi et al. (2021) | Yes | Yes | Yes | Yes | Yes | Yes | Yes | Yes | Yes | 9 | 100.00 |
| 118 | Sarna et al. (2021) | Yes | Yes | No | Yes | Yes | Yes | Yes | Yes | Yes | 8 | 88.89 |
| 119 | Siva et al. (2021) | Yes | No | No | Yes | Yes | Yes | Yes | Yes | Yes | 7 | 77.78 |
| 120 | Syamkumar et al. (2021) | Yes | Yes | No | Yes | Yes | Yes | Yes | Yes | Yes | 8 | 88.89 |
| 121 | Thapa et al. (2021) | Yes | Yes | Yes | Yes | Yes | Yes | Yes | Yes | Yes | 9 | 100.00 |
| 122 | Anam et al. (2022) | Yes | Yes | Yes | Yes | Yes | Yes | Yes | Yes | Yes | 9 | 100.00 |
| 123 | Arushi et al. (2022) | Yes | Unclear | Yes | No | Yes | Yes | Yes | Yes | Yes | 7 | 77.78 |
| 124 | Asghar et al. (2022) | Yes | Yes | Yes | No | Yes | Yes | Yes | Yes | Yes | 8 | 88.89 |
| 125 | Banerjee et al. (2022) | Yes | No | Yes | No | Yes | Yes | Yes | Yes | Yes | 7 | 77.78 |
| 126 | Bekhwani et al. (2022) | Yes | No | No | No | Yes | No | Yes | Yes | Yes | 5 | 55.56 |
| 127 | Bhusal et al. (2022) | Yes | Yes | Yes | Yes | Yes | Yes | Yes | Yes | Yes | 9 | 100.00 |
| 128 | Cazzaniga et al. (2022) | Yes | No | Yes | Yes | Yes | Yes | Yes | Yes | Yes | 8 | 88.89 |
| 129 | Chanchala et al. (2022) | Yes | No | Yes | No | Yes | Yes | Yes | Unclear | Yes | 6 | 66.67 |
| 130 | Dabas et al. (2022) | Yes | Yes | Yes | Yes | Yes | Yes | Yes | Yes | Yes | 9 | 100.00 |
| 131 | Ghayasuddin et al. (2022) | Yes | Yes | Yes | No | Yes | Yes | No | Yes | Yes | 7 | 77.78 |
| 132 | Jubayer et al. (2022) | Yes | Yes | Yes | Yes | Yes | Yes | Yes | Yes | Yes | 9 | 100.00 |
| 133 | Mogra et al. (2022) | Yes | No | Yes | No | Yes | Yes | Yes | Yes | Yes | 7 | 77.78 |
| 134 | Pradhan et al. (2022) | Yes | Yes | Yes | No | Yes | No | Yes | Yes | Yes | 7 | 77.78 |
| 135 | Tanveer et al. (2022) | Yes | Yes | Yes | Yes | Yes | Yes | Yes | Yes | Yes | 9 | 100.00 |
| 136 | Thilagar et al. (2022) | Yes | Yes | Yes | No | Yes | Yes | Yes | Yes | Yes | 8 | 88.89 |
| 137 | Uprety et al. (2022) | Yes | Yes | Yes | No | Yes | Yes | Yes | Yes | Yes | 8 | 88.89 |
| 138 | Yadawa et al. (2022) | Yes | No | Yes | No | Yes | Yes | Yes | Yes | Yes | 7 | 77.78 |
| 139 | Akthar et al. (2023) | Yes | No | No | No | Yes | Yes | Yes | Yes | Yes | 6 | 66.67 |
| 140 | Ashraf et al. (2023) | Yes | Yes | Yes | No | Yes | Yes | Yes | Yes | Yes | 8 | 88.89 |
| 141 | Bamania et al. (2023) | Yes | Yes | No | No | Yes | Yes | Yes | Yes | Yes | 7 | 77.78 |
| 142 | Bhattad et al. (2023) | Yes | No | Unclear | No | Yes | No | Yes | Yes | Yes | 5 | 55.56 |
| 143 | Dahal et al. (2023) | Yes | No | Yes | No | Yes | No | Yes | Yes | Yes | 6 | 66.67 |
| 144 | Hamann et al. (2023) | Yes | Yes | Yes | Yes | Yes | Yes | Yes | Yes | Yes | 9 | 100.00 |
| 145 | Herkar et al. (2023) | Yes | Yes | Yes | No | Yes | No | Yes | Yes | Yes | 7 | 77.78 |
| 146 | Hossain et al. (2023) | Yes | Yes | Unclear | Yes | Yes | Yes | Yes | Yes | Yes | 8 | 88.89 |
| 147 | Khatri et al. (2023) | Yes | Yes | Yes | No | Yes | Yes | Yes | Yes | Yes | 8 | 88.89 |
| 148 | Manna et al. (2023) | Yes | Yes | Yes | No | Yes | Yes | Yes | Yes | Yes | 8 | 88.89 |
| 149 | Rajnikanth et al. (2023) | Yes | Yes | Unclear | No | Yes | Yes | Yes | Yes | Yes | 7 | 77.78 |
| 150 | Santra et al. (2023) | Yes | Yes | Yes | No | Yes | Yes | Yes | Yes | Yes | 8 | 88.89 |
| 151 | Sivabalan et al. (2023) | Yes | Yes | Unclear | No | Yes | No | Yes | Yes | Yes | 6 | 66.67 |
| 152 | Sonar et al. (2023) | Yes | No | Yes | No | Yes | No | Yes | Yes | Yes | 6 | 66.67 |

**Supplemental Figure 1:** Funnel plot exhibiting publication bias for Overweight


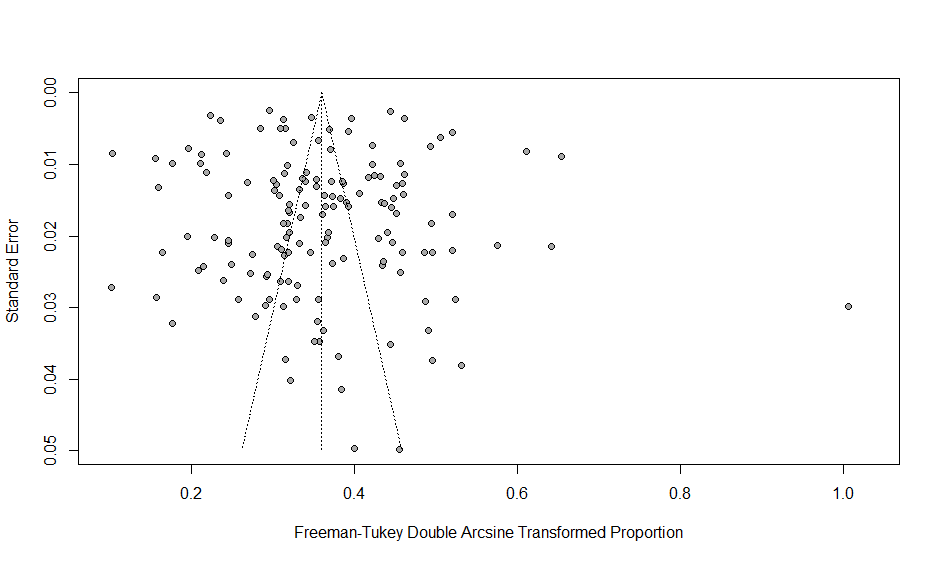


**Supplemental Figure 2:** Funnel plot exhibiting publication bias for OW+OB


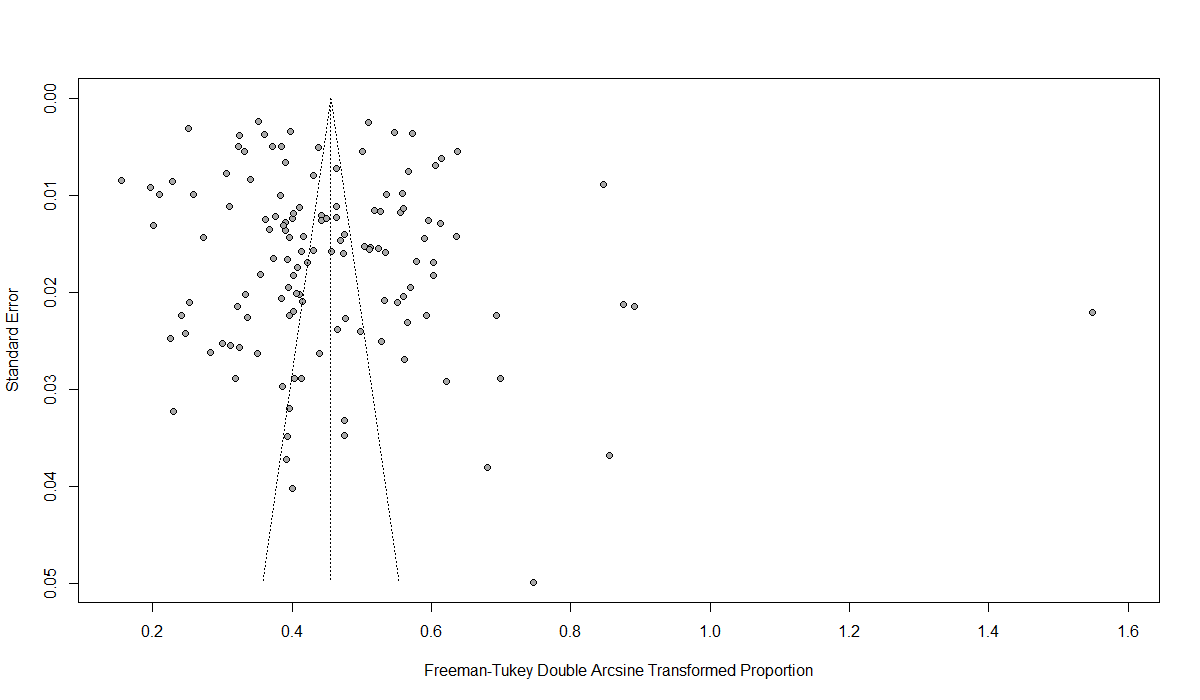


**Supplemental Figure 3:** Bubble plot exhibiting association of publication year and prevalence of OW


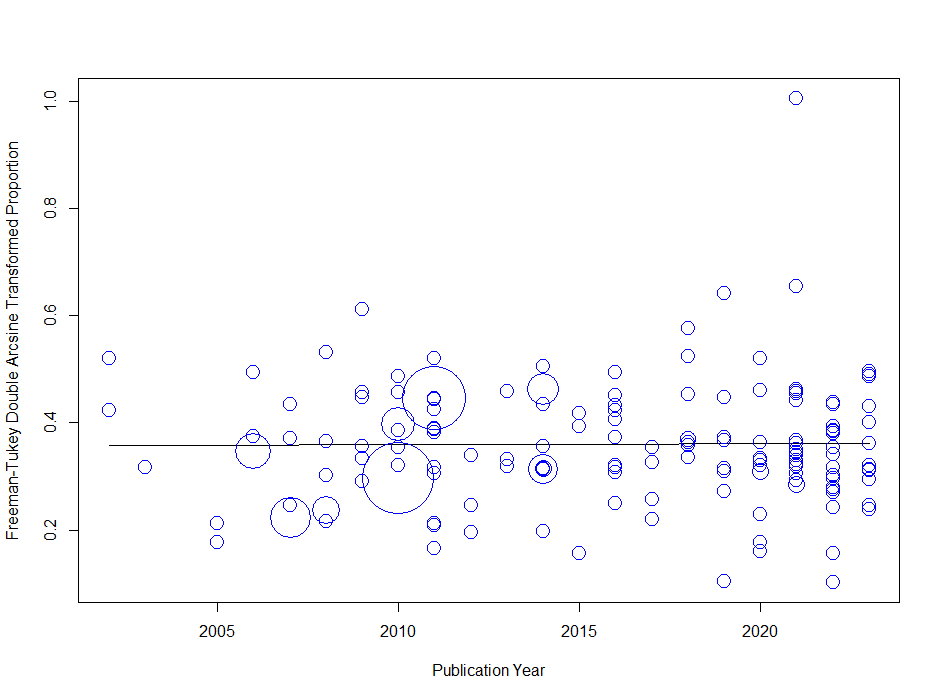


**Supplemental Figure 4:** Bubble plot exhibiting association of publication year and prevalence of OW+OB


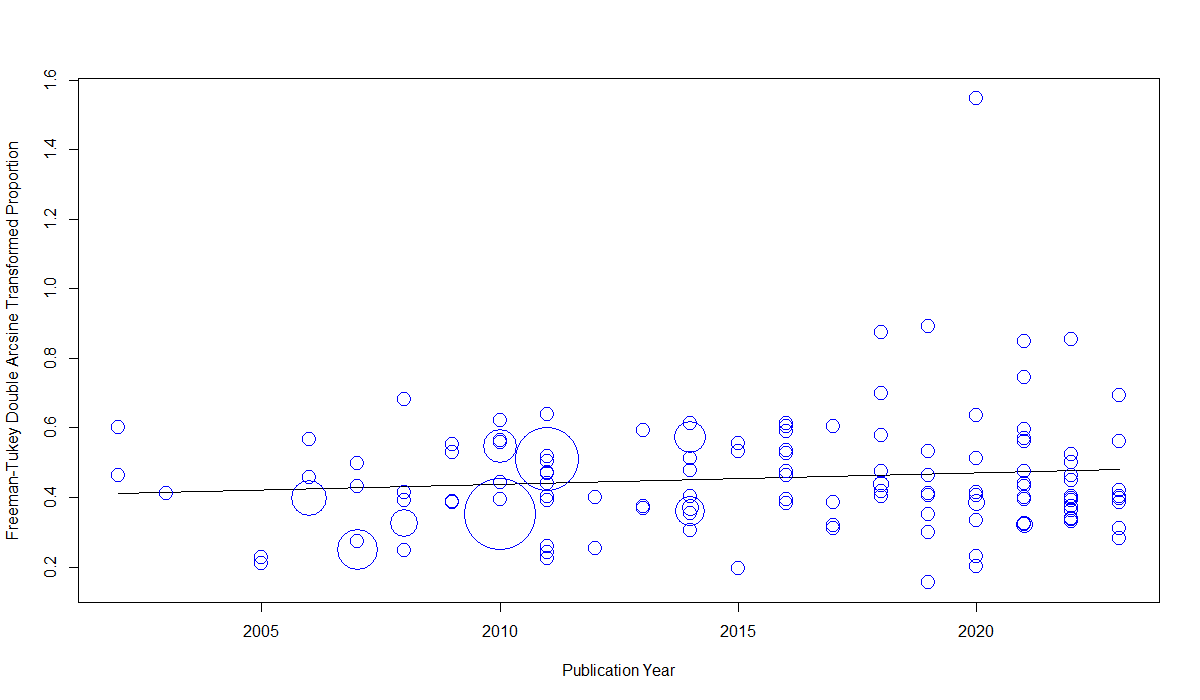


**References of Included Studies**

1. Verma M, Chhatwal J, George SM. Obesity and hypertension in children. Indian Pediatr. 1994;31(9):1065-9.

2. Kapil U, Singh P, Pathak P, Dwivedi SN, Bhasin S. Prevalence of obesity amongst affluent adolescent school children in delhi. Indian Pediatr. 2002;39(5):449-52.

3. Ramachandran A, Snehalatha C, Vinitha R, Thayyil M, Sathish Kumar CK, Sheeba L, et al. Prevalence of overweight in urban Indian adolescent school children. Diabetes Research and Clinical Practice. 2002;57(3):185-90.

4. Subramanyam V, Jayashree R, Rafi M. Prevalence of overweight and obesity in affluent adolescent girls in Chennai in 1981 and 1998. Indian Pediatr. 2003;40(8):775-9.

5. Marwaha RK, Tandon N, Singh Y, Aggarwal R, Grewal K, Mani K. A study of growth parameters and prevalence of overweight and obesity in school children from delhi. Indian Pediatr. 2006;43(11):943-52.

6. Sidhu S, Kaur N, Kaur R. Overweight and obesity in affluent school children of Punjab. Ann Hum Biol. 2006;33(2):255-9.

7. Sharma A, Sharma K, Mathur KP. Growth pattern and prevalence of obesity in affluent schoolchildren of Delhi. Public Health Nutr. 2007;10(5):485-91.

8. Bose K, Bisai S, Mukhopadhyay A, Bhadra M. Overweight and obesity among affluent Bengalee schoolgirls of Lake Town, Kolkata, India. Matern Child Nutr. 2007;3(2):141-5.

9. Laxmaiah A, Nagalla B, Vijayaraghavan K, Nair M. Factors affecting prevalence of overweight among 12- to 17-year-old urban adolescents in Hyderabad, India. Obesity (Silver Spring). 2007;15(6):1384-90.

10. Raj M, Sundaram KR, Paul M, Deepa AS, Kumar RK. Obesity in Indian children: time trends and relationship with hypertension. Natl Med J India. 2007;20(6):288-93.

11. Sood A, Sundararaj P, Sharma S, Kurpad AV, Muthayya S. BMI and body fat percent: affluent adolescent girls in Bangalore City. Indian Pediatr. 2007;44(8):587-91.

12. Aggarwal T, Bhatia RC, Singh D, Sobti PC. Prevalence of obesity and overweight in affluent adolescents from Ludhiana, Punjab. Indian Pediatr. 2008;45(6):500-2.

13. Kaur S, Sachdev HP, Dwivedi SN, Lakshmy R, Kapil U. Prevalence of overweight and obesity amongst school children in Delhi, India. Asia Pac J Clin Nutr. 2008;17(4):592-6.

14. Kumar HN, Mohanan P, Kotian S, Sajjan BS, Kumar SG. Prevalence of overweight and obesity among preschool children in semi urban South India. Indian Pediatr. 2008;45(6):497-49.

15. Ramzan M, Ali I, Khan AS. Body mass status of school children of Dera Ismail Khan, Pakistan. J Ayub Med Coll Abbottabad. 2008;20(4):119-21.

16. Sultan K, Habiba T. Prevalence of overweight and obesity in infancy. Bangladesh Med Res Counc Bull. 2008;34(2):69-70.

17. Bharati D, Deshmukh P, Garg B. Correlates of overweight & obesity among school going children of Wardha city, Central India. Indian Journal of Medical Research. 2008;127(6):539.

18. Pandit D, Chiplonkar S, Khadilkar A, Khadilkar V, Ekbote V. Body Fat Percentages by Dual-energy X-ray Absorptiometry Corresponding to Body Mass Index Cutoffs for Overweight and Obesity in Indian Children. Clin Med Pediatr. 2009;3:55-61.

19. Ghosh JR, Bandyopadhyay AR. Prevalence of thinness and overweight among urban adolescents of West Bengal, India. J Trop Pediatr. 2009;55(5):340-1.

20. Jeemon P, Prabhakaran D, Mohan V, Thankappan KR, Joshi PP, Ahmed F, et al. Double burden of underweight and overweight among children (10-19 years of age) of employees working in Indian industrial units. Natl Med J India. 2009;22(4):172-6.

21. Aziz S, Noorulain W, Zaidi UE, Hossain K, Siddiqui IA. Prevalence of overweight and obesity among children and adolescents of affluent schools in Karachi. J Pak Med Assoc. 2009;59(1):35-8.

22. Warraich HJ, Javed F, Faraz-Ul-Haq M, Khawaja FB, Saleem S. Prevalence of obesity in school-going children of Karachi. PLoS One. 2009;4(3):e4816.

23. Goyal RK, Shah VN, Saboo BD, Phatak SR, Shah NN, Gohel MC, et al. Prevalence of overweight and obesity in Indian adolescent school going children: its relationship with socioeconomic status and associated lifestyle factors. J Assoc Physicians India. 2010;58:151-8.

24. Bishwalata R, Singh AB, Singh AJ, Devi LU, Singh RK. Overweight and obesity among schoolchildren in Manipur, India. Natl Med J India. 2010;23(5):263-6.

25. Jain S, Pant B, Chopra H, Tiwari R. Obesity among adolescents of affluent public schools in Meerut. Indian J Public Health. 2010;54(3):158-60.

26. Kotian MS, S GK, Kotian SS. Prevalence and determinants of overweight and obesity among adolescent school children of South karnataka, India. Indian J Community Med. 2010;35(1):176-8.

27. Patnaik S, Patnaik L, Patnaik S, Hussain M. Prevalence of overweight and obesity in a private school of Orissa, India. Internet J Epidemiol. 2011;10(1):20-36.

28. Premanath M, Basavanagowdappa H, Shekar MA, Vikram SB, Narayanappa D. Mysore childhood obesity study. Indian Pediatr. 2010;47(2):171-3.

29. K R. Prevalence of overweight and obesity among high school students of Thiruvananthapuram City Corporation, Kerala, India. Australasian Medical Journal. 2010:650-61.

30. Anwar A, Anwar F, Joiya HU, Ijaz A, Rashid H, Javaid A, et al. Prevalence of obesity among the school-going children of Lahore and associated factors. J Ayub Med Coll Abbottabad. 2010;22(4):27-32.

31. ul Haq I, Siddiqui TS, Jan MA. Prevalence of obesity in school children of Hazara division. J Ayub Med Coll Abbottabad. 2010;22(4):50-2.

32. Khadilkar VV, Khadilkar AV, Cole TJ, Chiplonkar SA, Pandit D. Overweight and obesity prevalence and body mass index trends in Indian children. Int J Pediatr Obes. 2011;6(2-2):e216-24.

33. Sarkar M, Chatterjee S, Banerjee S. 74. Effect of Overweight and Obesity on Health-Related Quality of Life of Adolescents. Journal of Adolescent Health. 2011;48(2):S56.

34. Ferdousi J, Alamgir AKM, Hossain S, Karim R. Prevalence and determinants of overweight in school students: A developing country perspective. Canadian Journal of Diabetes. 2011;35.

35. Chakraborty P, Dey S, Pal R, Kar S, Zaman FA, Pal S. Obesity in Kolkata children: Magnitude in relationship to hypertension. J Nat Sci Biol Med. 2011;2(1):101-6.

36. Ghosh A. Rural-urban comparison in prevalence of overweight and obesity among children and adolescents of Asian Indian origin. Asia Pac J Public Health. 2011;23(6):928-35.

37. Goyal JP, Kumar N, Parmar I, Shah VB, Patel B. Determinants of Overweight and Obesity in Affluent Adolescent in Surat City, South Gujarat region, India. Indian J Community Med. 2011;36(4):296-300.

38. Gupta DK, Shah P, Misra A, Bharadwaj S, Gulati S, Gupta N, et al. Secular trends in prevalence of overweight and obesity from 2006 to 2009 in urban asian Indian adolescents aged 14-17 years. PLoS One. 2011;6(2):e17221.

39. Prashanth K, Baby KE, Rao KR, Kumarkrishna B, Hegde K, Kumar M, et al. Prevalence of obesity among high school children in Dakshina Kannada and Udupi districts. Journal of Health and Allied Sciences NU. 2011;1(04):16-20.

40. Mahajan PB, Purty AJ, Singh Z, Cherian J, Natesan M, Arepally S, et al. Study of childhood obesity among school children aged 6 to 12 years in union territory of puducherry. Indian J Community Med. 2011;36(1):45-50.

41. Misra A, Shah P, Goel K, Hazra DK, Gupta R, Seth P, et al. The high burden of obesity and abdominal obesity in urban Indian schoolchildren: a multicentric study of 38,296 children. Ann Nutr Metab. 2011;58(3):203-11.

42. Thakre SB, Mohane SP, Ughade SM, Thakre SS, Morey SS, Humne AY. Correlates of overweight and obesity among urban school going children of Nagpur city. Journal of Clinical and Diagnostic Research. 2011;5:1593-7.

43. Iyer U, Kumar N, Akolkar A. Magnitude and Determinants of Overweight and Obesity in 6-12 year old school children of Vadodara City. Curr Pediatr Res. 2011;15.

44. Vohra R, Bhardwaj P, Srivastava JP, Srivastava S, Vohra A. Overweight and obesity among school-going children of Lucknow city. J Family Community Med. 2011;18(2):59-62.

45. Mushtaq MU, Gull S, Abdullah HM, Shahid U, Shad MA, Akram J. Prevalence and socioeconomic correlates of overweight and obesity among Pakistani primary school children. BMC Public Health. 2011;11:724.

46. Deoke A, Hajare S, Saoji A. Prevalence of overweight in high school students with special reference to cardiovascular efficiency. Glob J Health Sci. 2012;4(2):147-52.

47. Sarkar SR, Saha S, Roy S, Sil SK. Nutritional status of Tripuri tribal adolescent boys of West Tripura district. Indian Pediatr. 2012;49(6):494-5.

48. Cherian AT, Cherian SS, Subbiah S. Prevalence of obesity and overweight in urban school children in Kerala, India. Indian Pediatr. 2012;49(6):475-7.

49. Maiti S, De D, Ali KM, Bera TK, Ghosh D, Paul S. Overweight and obesity among early adolescent school girls in urban area of west bengal, India: prevalence assessment using different reference standards. Int J Prev Med. 2013;4(9):1070-4.

50. Wickramasinghe VP, Arambepola C, Bandara P, Abeysekera M, Kuruppu S, Dilshan P, et al. Distribution of obesity-related metabolic markers among 5-15 year old children from an urban area of Sri Lanka. Ann Hum Biol. 2013;40(2):168-74.

51. Hashmi A, Soomro JA, Saleem K. Prevalence of obesity and factors leading to obesity among high school students of Pakistan. Journal of Medicine. 2013;14(1):33-9.

52. George GM, Sharma KK, Ramakrishnan S, Gupta SK. A study of cardiovascular risk factors and its knowledge among school children of Delhi. Indian Heart J. 2014;66(3):263-71.

53. Jagadesan S, Harish R, Miranda P, Unnikrishnan R, Anjana RM, Mohan V. Prevalence of overweight and obesity among school children and adolescents in Chennai. Indian pediatrics. 2014;51(7):544-9.

54. Kajale NA, Khadilkar AV, Chiplonkar SA, Khadilkar VV. Body fat indices for identifying risk of hypertension in Indian children. Indian Pediatr. 2014;51(7):555-60.

55. Gurung TR, Neginhal V. Overweight and obesity among the adolescent school students in Belgaum city. J Nepal Med Assoc. 2014;52(194):791-5.

56. Parashar P, Bansal R, Sharma S, Varshney AM, Shukla A, Ahmad S. Body Mass Index–for–Age Criteria: a School Based Study in Meerut UP. EXECUTIVE EDITOR. 2015;6(4):151.

57. Aroor AR, Airody SK, Mahale R, Sr R, Shetty S, Rao AR. Anthropometry and Prevalence of Common Health Problems among School Going Children in Surathkal, Karnataka. J Clin Diagn Res. 2014;8(12):PC01-5.

58. Kumaravel V, Shriraam V, Anitharani M, Mahadevan S, Balamurugan AN, Sathiyasekaran BW. Are the current Indian growth charts really representative? Analysis of anthropometric assessment of school children in a South Indian district. Indian J Endocrinol Metab. 2014;18(1):56-62.

59. Ghosh A. Explaining overweight and obesity in children and adolescents of Asian Indian origin: the Calcutta childhood obesity study. Indian J Public Health. 2014;58(2):125-8.

60. Mithra PP, Kumar P, Kamath VG, Kamath A, Unnikrishnan B, Rekha T, et al. Lifestyle factors and obesity among adolescents in rural South India. Asian Journal of Pharmaceutical and Clinical Research. 2015;8(6):81-3.

61. Bulbul T, Hoque M. Prevalence of childhood obesity and overweight in Bangladesh: findings from a countrywide epidemiological study. BMC Pediatr. 2014;14:86.

62. Patnaik L, Pattanaik S, Sahu T, Venkata Rao E. Overweight and obesity among adolescents–A comparative study between government and private schools. Indian pediatrics. 2015;52(9):779-81.

63. Koirala M, Khatri RB, Khanal V, Amatya A. Prevalence and factors associated with childhood overweight/obesity of private school children in Nepal. Obes Res Clin Pract. 2015;9(3):220-7.

64. Goel M, Pal P, Agrawal A, Ashok C. Relationship of body mass index and other life style factors with hypertension in adolescents. Ann Pediatr Cardiol. 2016;9(1):29-34.

65. Saikia D, Ahmed SJ, Saikia H, Sarma R. Overweight and obesity in early adolescents and its relation to dietary habit and physical activity: A study in Dibrugarh town. Clinical Epidemiology and Global Health. 2016;4:S22-S8.

66. Eshwar TK, Chudasama RK, Eshwar ST, Thakrar D. Prevalence of obesity and overweight and their comparison by three growth standards among affluent school students aged 8-18 years in Rajkot. Indian J Public Health. 2017;61(1):51-4.

67. Prasad R, Bazroy J, Singh Z. Prevalence of overweight and obesity among adolescent students in Pondicherry, South India. International Journal of Nutrition, Pharmacology, Neurological Diseases. 2016;6(2).

68. Pawar SV, Choksey AS, Jain SS, Surude RG, Rathi PM. Prevalence of Overweight and Obesity in 4 Schools of South Mumbai. J Clin Diagn Res. 2016;10(3):OC01-2.

69. Aluckal E, Anzil K, Baby M, George EK, Lakshmanan S, Chikkanna S. Association between Body Mass Index and Dental Caries among Anganwadi Children of Belgaum City, India. J Contemp Dent Pract. 2016;17(10):844-8.

70. Bhargava M, Kandpal SD, Aggarwal P, Sati HC. Overweight and Obesity in School Children of a Hill State in North India: Is the Dichotomy Urban-Rural or Socio-Economic? Results from a Cross-Sectional Survey. PLoS One. 2016;11(5):e0156283.

71. Hussain M, Tenglikar P, Nigudgi S. Physical activity and its association with body mass index among 10-15 years school children in Kalaburagi city, Karnataka, India. International Journal of Community Medicine and Public Health. 2016:2264-9.

72 Rexlin GB, Sivakumar E, Rajkumar DD, Nagendran MV. Prevalence of obesity among school children in madurai. International journal of current research and review. 2016;8:01-6

73. Nawab T, Khan Z, Khan IM, Ansari MA. Is small town India falling into the nutritional trap of metro cities? A study in school-going adolescents. J Family Med Prim Care. 2016;5(3):581-6.

74. Shuhana S. Prevalence and risk factor of childhood overweight and obesity in primary school children of Dhaka city: Det medisinske fakultet, Universitetet i Oslo; 2010.

75. Bharati S, Pal M, Shome S, Roy P, Dhara P, Bharati P. Influence of socio-economic status and television watching on childhood obesity in Kolkata. Homo. 2017;68(6):487-94.

76. Mishra A, Acharya H. Factors influencing obesity among school-going children in Sambalpur district of Odisha. Journal of Medical Society. 2017;31(3).

77. Ganie MA, Bhat GA, Wani IA, Rashid A, Zargar SA, Charoo BA, et al. Prevalence, risk factors and consequences of overweight and obesity among schoolchildren: a cross-sectional study in Kashmir, India. J Pediatr Endocrinol Metab. 2017;30(2):203-9.

78. Faizi N, Khan Z, Khan IM, Amir A, Azmi SA, Khalique N. A study on nutritional status of school-going adolescents in Aligarh, India. Trop Doct. 2017;47(3):212-6.

79. Greene-Cramer B, Harrell MB, Hoelscher DM, Sharma S, Ranjit N, Gupta V, et al. Association between parent and child weight status among private school children in Delhi, India. Glob Health Promot. 2018;25(2):67-74.

80. Shete JS, Wagh AV. A cross sectional study to estimate prevalence of obesity and its risk factors in adolescent school children in Western Maharashtra, India. International Journal of Research in Medical Sciences. 2018;6(9).

81. Kuriyan R, Selvan S, Thomas T, Jayakumar J, Lokesh DP, Phillip MP, et al. Body Composition Percentiles in Urban South Indian Children and Adolescents. Obesity (Silver Spring). 2018;26(10):1629-36.

82. Halder S, Kaul R, Angrish P, Saha S, Bhattacharya B, Mitra M. Association between obesity and oral health status in schoolchildren: a survey in five districts of West Bengal, India. International Journal of Clinical Pediatric Dentistry. 2018;11(3):233.

83. Mansoori N, Nisar N, Shahid N, Mubeen SM, Ahsan S. Prevalence of obesity and its risk factors among school children in Karachi, Pakistan. Trop Doct. 2018;48(4):266-9.

84. Sajon S, Aziz, Sana S, Rana S, Atikullah, Akter, et al. Prevalence of overweight and obesity among urban school children and adolescents in Khulna, Bangladesh. Pharmacologyonline. 2018;1.

85. Sultana N, Afroz S, Tomalika N, Momtaz H, Kabir MH. Prevalence of Childhood Obesity and Undernutrition among Urban School Children in Bangladesh. J Biosoc Sci. 2019;51(2):244-53.

86. Mohan B, Verma A, Singh K, Singh K, Sharma S, Bansal R, et al. Prevalence of sustained hypertension and obesity among urban and rural adolescents: a school-based, cross-sectional study in North India. BMJ Open. 2019;9(9):e027134.

87. Chandra N, Anne B, Venkatesh K, Teja GD, Katkam SK. Prevalence of Childhood Obesity in an Affluent School in Telangana Using the Recent IAP Growth Chart: A Pilot Study. Indian J Endocrinol Metab. 2019;23(4):428-32.

88. Dolkar T, Mehta B, Wangdi J. A Study on the Nutritional Status of the School Going Adolescents of East Sikkim, North East India. Indian Journal of Public Health Research & Development. 2019;10:232.

89. Dabade S, Dabade K. Prevalence of overweight and obesity among school-going children of Satara district, Maharashtra. National Journal of Physiology, Pharmacy and Pharmacology. 2019(0).

90. Khan MMA, Karim M, Islam AZ, Islam MR, Khan HTA, Khalilullah MI. Prevalence of overweight and obesity among adolescents in Bangladesh: do eating habits and physical activity have a gender differential effect? J Biosoc Sci. 2019;51(6):843-56.

91. Karki A, Shrestha A, Subedi N. Prevalence and associated factors of childhood overweight/obesity among primary school children in urban Nepal. BMC Public Health. 2019;19(1):1055.

92. Manandhar S, Suksaroj TT, Rattanapan C. The Association between Green Space and the Prevalence of Overweight/Obesity among Primary School Children. Int J Occup Environ Med. 2019;10(1):1-10.

93. Norbu W, Wangdi U, Dorji D, Arthan D, Soonthornworasiri N, Maneekan P, et al. Obesity prevalence and contributing factors among adolescents in secondary schools in Pemagatshel district, Bhutan. Int J Adolesc Med Health. 2017;31(1).

94. Daga S, Mhatre S, Kasbe A, Dsouza E. Double burden of malnutrition among Indian schoolchildren and its measurement: a cross-sectional study in a single school. BMJ Paediatr Open. 2020;4(1):e000505.

95. Kaur S, Chandel S, Chandel S. The relationship between body mass index and blood pressure: A study among school going children of Rajasthan, India. Indian Journal of Physiology and Pharmacology. 2021;64:236-41.

96. Singh P, Gandhi S, Malhotra RK, Seth A. Impact of Using Different Growth References on Interpretation of Anthropometric Parameters of Children Aged 8-15 Years. Indian Pediatr. 2020;57(2):124-8.

97. Kavi A, Walvekar PR. Lifestyle factors influencing the academic performance among the secondary school students in an urban area of south India. Int J Adolesc Med Health. 2020.

98. Viswanathan VT, Patil SS, Durgawale P, Kakade S. Study of Prevalence and Lifestyle Related Correlates of Overweight and Obesity Among Rural Adolescents of Western Maharashtra. International Journal of Nutrition, Pharmacology, Neurological Diseases. 2020;10(2):29.

99. Bhuvaneswari B, Parameshwari S. Potential Factors related to BMI among School going adolescents of Madurai District, Tamil Nadu, India. International Journal of Research in Pharmaceutical Sciences. 2020;11(4):5448-52.

100. Solanki DK, Walia R, Gautam A, Misra A, Aggarwal AK, Bhansali A. Prevalence of abdominal obesity in non-obese adolescents: a North Indian adolescent study. J Pediatr Endocrinol Metab. 2020;33(7):853-8.

101. Naskar P, Roy S. BMI and Lifestyle Pattern - A Cross Sectional Study among Adolescent School Students in an Urban Area of West Bengal, India. Journal of Evidence Based Medicine and Healthcare. 2020;7(46):2719-23.

102. Hassan MT, Das H, Banik S. A cross-sectional study to determine the prevalence of overweight and obesity among Bangladeshi adolescents based on WHO, IOTF, and CDC cut-points. Obesity Medicine. 2020;19.

103. Singh DP, Arya A, Kondepudi KK, Bishnoi M, Boparai RK. Prevalence and associated factors of overweight/obesity among school going children in Chandigarh, India. Child: Care, Health and Development. 2020;46(5):571-5.

104. Tariq S, Tariq S, Tariq S, Rehman R. Relationship of BMI with Junk Food, sleep pattern, exam performance and awareness about its ill health effects in healthy teenagers. J Pak Med Assoc. 2021;71(1(A)):59-63.

105. Sathiadas MG, Antonyraja A, Viswalingam A, Thangaraja K, Wickramasinghe VP. Nutritional status of school children living in Northern part of Sri Lanka. BMC Pediatr. 2021;21(1):43.

106. Bhat A, Sharma M. Prevalence of obesity and hypertension among teenage girls in an emerging metropolitan city of Central Rajasthan. National Journal of Physiology, Pharmacy and Pharmacology. 2021(0).

107. Bodhare T, Bele S, Murugvel H, Anto JV. Prevalence and Risk Factors of Hypertension, Overweight and Obesity among School Children in Madurai, Tamil Nadu: A Cross Sectional Study. Journal of Krishna Institute of Medical Sciences (JKIMSU). 2021;10(3).

108. Das RR, Mangaraj M, Nayak S, Satapathy AK, Mahapatro S, Goyal JP. Prevalence of Insulin Resistance in Urban Indian School Children Who Are Overweight/Obese: A Cross-Sectional Study. Front Med (Lausanne). 2021;8:613594.

109. Malarvizhi D, Devika R. Association of Physical Activity With Flexibility in Overweight and Obese Children. International Journal of Research in Pharmaceutical Sciences. 2021;12(1):46-51.

110. Mangalam K, Muneesh C, Akshay A, Ambreen F, Pramod S. Prevalence of Overweight and Obesity among School Going Adolescent in Patna. Medico-Legal Updat. 2021;21(1):446-50.

111. Mathew S, Dsouza J, Saldanha P. Lifestyle Practices and Knowledge on Prevention and Control of Overweight and Obesity among Adolescents: A Cross-sectional Study. Journal of Clinical and Diagnostic Research. 2021

112. Moudgil S, Mahajan R, Narang T, Sachdeva N, Dayal D, Dogra S. Central obesity and dyslipidemia in pediatric patients with psoriasis: An observational study from India. J Am Acad Dermatol. 2021;85(6):1655-7.

113. PALADUGU N, RAYITI R, NUTAKKI S, GADDE R, GHANTA VK, KANAGALA VS. Factors associated with body mass index in children–a community-based study. Asian Journal of Pharmaceutical and Clinical Research. 2021:176-81.

114. Qaisar R, Karim A. BMI status relative to international and national growth references among Pakistani school-age girls. BMC Pediatr. 2021;21(1):535.

115 Rawal T, Willeboordse M, Arora M, Sharma N, Nazar GP, Tandon N, van Schayck CP. Prevalence of Excessive Weight and Underweight and Its Associated Knowledge and Lifestyle Behaviors among Urban Private School-Going Adolescents in New Delhi. Nutrients. 2021 Sep 21;13(9):3296. doi: 10.3390/nu13093296.

116. Saeedullah A, Khan MS, Andrews SC, Iqbal K, Ul-Haq Z, Qadir SA, et al. Nutritional Status of Adolescent Afghan Refugees Living in Peshawar, Pakistan. Nutrients. 2021;13(9).

117. Salvi SS, Kumar A, Puri H, Bishnoi S, Asaf BB, Ghorpade D, et al. Association between air pollution, body mass index, respiratory symptoms, and asthma among adolescent school children living in Delhi, India. Lung India. 2021;38(5):408-15.

118. Sarna A, Porwal A, Acharya R, Ashraf S, Ramesh S, Khan N, et al. Waist circumference, waist-to-height ratio and BMI percentiles in children aged 5 to 19 years in India: A population-based study. Obes Sci Pract. 2021;7(4):392-404.

119. Siva L, Krishnamoorthy V, Durai KS, Ahamed SSS, Rajakumari S, Catherine N. Comparative evaluation of body mass index among school children with and without Bruxism of age group of 6–12 years in Kanchipuram district: A cross-sectional study. Journal of Indian Society of Pedodontics and Preventive Dentistry. 2021;39(1):42.

120. Syamkumar V, Thomas AJ, Oommen S, Aswin S, Anand PS, Mathew V. Relationship between body mass index and dental caries in 13–15-year-old school children of government and private schools in Bengaluru City. Journal of Pharmacy & Bioallied Sciences. 2021;13(Suppl 1):S841.

121. Thapa A, Nepal S, Malla G, Pokhrel S. Childhood Overnutrition among School Going Children in a Municipality: A Descriptive Cross-sectional Study. JNMA J Nepal Med Assoc. 2021;59(242):1004-7.

122. Anam MR, Akter S, Hossain F, Bonny SQ, Akter J, Zhang C, et al. Association of sleep duration and sleep quality with overweight/obesity among adolescents of Bangladesh: a multilevel analysis. BMC Public Health. 2022;22(1):374.

123. Arushi P, Dileep G, Devendra S, Hemlata M, Pratee A. Effect of Screen Time, Hours of Physical Activity and Sleep Hours on Overweight and Obesity in School-Going Adolescent Girls of Udaipur. International Journal of Toxicological and Pharmacological Research. 2022;12(6):17-27.

124. Asghar M, Amung R, Chakrabarty S. Nutritional Status of Children (5-18 Years) by Using Anthropometric Indices: A Cross-Sectional Study Among the Sartang and Miji, Lesser Known Tribes of Arunachal Pradesh, India. Journal of Nepal Paediatric Society. 2022;41(3):413-8.

125. Banerjee M, Bhatti BVK, Roy D, Tomo S. A National Survey of the Prevalence of Anemia and Obesity in Indian School Children. J Community Hosp Intern Med Perspect. 2022;12(5):48-53.

126. Bekhwani AR, Khan M. Various Risk Factors of Overweight and Obesity among Children Aged 5-16 Years. J Coll Physicians Surg Pak. 2022;32(6):763-7.

127. Bhusal CK, Bhattarai S, Chhetri P, Myia SD. Nutritional status and its associated factors among under five years Muslim children of Kapilvastu district, Nepal. PLoS One. 2023;18(1):e0280375.

128. Cazzaniga E, Orlando A, Terenzio A, Suardi C, Mognetti C, Gennaro F, et al. Health Status and Nutritional Habits in Maldives Pediatric Population: A Cross-Sectional Study. Int J Environ Res Public Health. 2022;19(23).

129. Chanchala HP, Madhu B, Nagaraja MS, Shanbhog R. Secular trends in prevalence of overweight and obesity over a decade in urban and rural South Indian children integrated with geographic information system. Indian J Dent Res. 2022;33(3):235-40.

130. Dabas A, Rastogi V, Khadgawat R, Marwaha RK. Predictive Performance of Different Diagnostic Criteria for Overweight and Obesity Between 2008-2015 in Adolescents. Indian Pediatr. 2022;59(2):110-3.

131. Ghayasuddin S, Ali W, Khalil KUR, Ali I, Ahmad S. OVERWEIGHT, OBESITY AND ITS ASSOCIATED RISK FACTORS AMONG SCHOOL GOING CHILDREN OF PESHAWAR, KHYBER PAKHTUNKHWA, PAKISTAN. Journal of Medical Sciences (Peshawar). 2022;30(4):250-5.

132. Jubayer A, Islam MH, Nayan MM. Malnutrition among under-five children in St. Martin's Island, Bangladesh: A cross-sectional study on prevalence and associated factors. SAGE Open Med. 2022;10:20503121221116246.

133. Mogra G, Arora KK, Saroha R, Kela G. PREVALENCE OF HYPERTENSION AND ITS ASSOCIATION WITH OBESITY AMONG SCHOOL GOING CHILDREN IN SELECTED SCHOOLS OF INDORE. Journal of Cardiovascular Disease Research. 2022;13(5):2626-31.

134. Pradhan S, Beriha SS, Patjoshi SK, Supakar S. PREVALENCE OF OBESITY AMONG ADOLESCENT SCHOOL CHILDREN IN RURAL AND URBAN SOUTH ODISHA. International Journal of Academic Medicine and Pharmacy. 2022;4(3):261-5.

135. Tanveer M, Hohmann A, Roy N, Zeba A, Tanveer U, Siener M. The Current Prevalence of Underweight, Overweight, and Obesity Associated with Demographic Factors among Pakistan School-Aged Children and Adolescents-An Empirical Cross-Sectional Study. Int J Environ Res Public Health. 2022;19(18).

136. Thilagar M, Tiwari P. Burden of Overweight and Obesity Among Secondary and Senior Secondary Students of New Delhi Municipal Council Schools. Indian Journal of Public Health Research and Development. 2022;13(4):100-5.

137. Uprety S, Khadka A, Paudyal A, Shrestha D. Nutritional Status and Practices among Urban Children: A Hospital-based Cross-sectional Study. J Nepal Health Res Counc. 2022;20(2):331-8.

138. Yadawa VK, Saroj KS, Prasad R. Study to evaluate the risk factors of overweight and obesity among higher secondary school children in Muzaffarpur, Bihar. Indian Journal of Public Health Research and Development. 2022;13(1):412-7.

139. Akhtar N, Zainab S, Sualeh Z, Arif H, Sualeh MA, Abeer SJ. Prevalence and Factors Associated with Growth Patterns of School Children in K hi. Medical Forum Monthly. 2023;34(1):25-8.

140. Ashraf S, Mushtaq S, Zahra FT, Siddiqa S, Quratulain, Bano N. Nutritional Status of School-Going Children Visiting Out Patient Department of a Tertiary Care Hospital. Pakistan Journal of Medical and Health Sciences. 2023;17(3):110-2.

141. Bamania M, Banker K, Patel N, Menat SK. A study on assessment of correlation between obesity and blood groups among school children of Gujarat, India. National Journal of Physiology, Pharmacy and Pharmacology. 2023;13(5):1046-9.

142. Bhattad S, Rathi SA, Kendre V, Bhangdiya SO, Takalkar AA. Assessment Of BMI In School Going Children And Its Association With Some Sociodemographic Factors. Journal of Cardiovascular Disease Research. 2023;14(7):925-30.

143. Dahal D, Amita KC, Chand S, Pant RD, Dikkatwar MS, Pant BD. Determinants of Nutritional Status among Mothers and their Children of Age 6-59 Months. Global Journal of Medical Pharmaceutical and Biomedical Update. 2023;18.

144. Hamann SA, Thorup L, Patsche CB, Hohwü L, Hjortdal VE, Gyawali B, et al. Association between nutritional status and socio-economic status among school children aged 9-17 years in a semi-urban area of Nepal. J Health Popul Nutr. 2023;42(1):53.

145. Herkar RS, Patil KR, Kabra YM. To Study the prevalence of overweight and obesity in school children A Cross sectional study. Journal of Cardiovascular Disease Research. 2023;14(1):418-24.

146. Hossain MS, Raheem E, Okely AD. 24-hour movement guidelines and weight status among preschool-aged children in Bangladesh: A community-level cross-sectional study. Brain Behav. 2023;13(7):e3094.31.

147. Khatri E, Baral K, Arjyal A, Yadav RK, Baral S. Prevalence of and risk factors for overweight among adolescents of a sub-metropolitan city of Nepal. PLoS One. 2023;18(3):e0270777.

148. Manna N, Banerjee S, Panchanan P, Mazumdar SD. An epidemiological study on junk food consumption and nutritional status among adolescents attending Medical College and Hospital, Kolkata. National Journal of Physiology, Pharmacy and Pharmacology. 2023;13(7):1554-8.

149. Rajinikanth BS, U S, Yadav S. Prevalence of Obesity and Its Relationship With Hypertension Among School-Going Adolescents Aged 12-16 Years. Cureus. 2023;15(8):e42999.

150. Santra A, Rai S, Misra P, Yadav K, Goswami K, Kaur G. Comparison of Different Anthropometric Indicators for Assessment of Nutritional Status Among Adolescent Girls in an Urban Resettlement Colony in New Delhi: A Cross-Sectional Study. Cureus. 2023;15(4):e37242.

151. Sivabalan T, Chakkaravarthy K, Naveen G, Niaz MAM. Analyzing the rates of obesity, hypertension, and their risk factors among 12-16 year school children in Perambalur district. Journal of Cardiovascular Disease Research. 2023;14(4):333-8.

152. Sonar M, Mashalkar D, Astha A, Ray S. Prevalence of Childhood Obesity and Its Association with Socioeconomic Factors: A Cross-Sectional Study. Journal of Cardiovascular Disease Research. 2023;14(7):793-800.
